# Biophysical Insights into How Spike Threshold Depends on the Rate of Membrane Potential Depolarization in Type I and Type II Neurons

**DOI:** 10.1371/journal.pone.0130250

**Published:** 2015-06-17

**Authors:** Guo-Sheng Yi, Jiang Wang, Kai-Ming Tsang, Xi-Le Wei, Bin Deng

**Affiliations:** 1 School of Electrical Engineering and Automation, Tianjin University, Tianjin, China; 2 Department of Electrical Engineering, The Hong Kong Polytechnic University, Hong Kong, China; McGill University, CANADA

## Abstract

Dynamic spike threshold plays a critical role in neuronal input-output relations. In many neurons, the threshold potential depends on the rate of membrane potential depolarization (d*V*/d*t*) preceding a spike. There are two basic classes of neural excitability, i.e., Type I and Type II, according to input-output properties. Although the dynamical and biophysical basis of their spike initiation has been established, the spike threshold dynamic for each cell type has not been well described. Here, we use a biophysical model to investigate how spike threshold depends on d*V*/d*t* in two types of neuron. It is observed that Type II spike threshold is more depolarized and more sensitive to d*V*/d*t* than Type I. With phase plane analysis, we show that each threshold dynamic arises from the different separatrix and K+ current kinetics. By analyzing subthreshold properties of membrane currents, we find the activation of hyperpolarizing current prior to spike initiation is a major factor that regulates the threshold dynamics. The outward K+ current in Type I neuron does not activate at the perithresholds, which makes its spike threshold insensitive to d*V*/d*t*. The Type II K+ current activates prior to spike initiation and there is a large net hyperpolarizing current at the perithresholds, which results in a depolarized threshold as well as a pronounced threshold dynamic. These predictions are further attested in several other functionally equivalent cases of neural excitability. Our study provides a fundamental description about how intrinsic biophysical properties contribute to the threshold dynamics in Type I and Type II neurons, which could decipher their significant functions in neural coding.

## Introduction

Neurons encode and propagate information by transforming various spatiotemporal patterns of synaptic input into sequences of action potentials or spikes [1, 2], which are usually regarded as the principal carrier of information. The action potential can be evoked when membrane depolarization reaches a threshold level. This is a special membrane potential value that distinguishes suprathreshold depolarization from subthreshold [2]. The spike threshold is a basic biophysical property for all spiking neurons, which functions as a high-pass filter and plays a crucial role in action potential initiation [2–5].

Many experiment recordings *in vivo* have shown that the spike threshold is not constant but dynamic, which varies with neuronal recent spiking activities and synaptic inputs [2–16]. Particularly, spike threshold is dependent on the membrane potential changes, such as the rate of membrane potential depolarization (i.e., d*V*/d*t*) preceding spike initiation, which has been observed in many areas of the central nervous system [4–10, 12–16]. The threshold variability dependent on d*V*/d*t* could be regulated by the intrinsic properties of membrane currents, especially Na^+^ channel inactivation [3, 6–8, 17–20] and K^+^ channel activation [4, 11, 20, 21–24]. The dynamic changes of spike threshold would have a profound influence on neural input-output properties, such as, enhancing feature selectivity [7, 8, 14, 25, 26], contributing to neuron sensitivity [6–8, 12, 15], improving coincidence detection and gain modulation [3, 6, 7, 27], as well as facilitating precise temporal coding [4, 20, 28].

According to the input-output relations, there is one basic classification of neurons [2, 29–31], i.e., Type I and Type II (sometimes called Class 1 and Class 2) excitabilities, which is first identified by Hodgkin. For Type I neurons, they have a continuous frequency-current (*f*-*I*) curve, and their spike initiation dynamic is characterized by a saddle-node on invariant circle (SNIC) bifurcation. Unlike Type I, the Type II neurons have a discontinuous *f*-*I* curve and their spike initiation dynamic is characterized by a Hopf bifurcation. It has been shown that the distinct spike initiation dynamics associated with each cell type are also the different outcomes of the nonlinear competition between inward (depolarizing) and outward (hyperpolarizing) membrane currents [29]. In Type I neurons, their outward K^+^ current activates at a higher potential than inward Na^+^ current. In this case, the Na^+^ current could depolarize membrane potential without competition and drive it slowly across threshold. Thus, the Type I neuron is able to generate arbitrary low frequency spikes to weak current stimulus. However, the outward K^+^ current has activated at the perithreshold potentials in Type II neurons. Their spike initiation occurs when inward Na^+^ current activates faster than K^+^ current to drive membrane potential through threshold with sufficient speed. Thus, they fail to maintain repetitive spiking at low rates. The different spike initiation mechanisms for two types of neurons endow them with distinct coding capabilities to stimulus [2, 29–31]. Despite the dynamical and biophysical basis for Type I and Type II excitabilities has been established, the relation between their spike initiation and spike threshold dynamics is still not well described. Only one previous investigation by Izhikevich [2, 32] shows that Type I neuron has a well-defined threshold manifold in phase space, whereas Type II does not have. Hence, there are still many important questions unanswered. How does spike threshold depends on d*V*/d*t* in Type I and Type II neurons? Could their different spike initiation mechanisms also result in distinct spike threshold dynamics? If so, what is the underlying mechanism? How do the intrinsic properties of membrane currents in two cell types contribute to their threshold dynamics?

To address these questions, we adopt a quantitative approach proposed by Wester and Contreras [20] to determine the spike threshold within a range of d*V*/d*t* for Type I and Type II neuron models. We find that Type II neuron has a more depolarized threshold than Type I. Meanwhile, its spike threshold is sensitive to d*V*/d*t*, while Type I is insensitive. By determining the threshold manifold in phase space, we explain why they have distinct changes in spike threshold as d*V*/d*t* increases. The approach for determining spike threshold allows us to observe the membrane currents and their gating kinetics at the threshold potentials, through which we could identify how the intrinsic properties of ionic currents contribute to the relationship between spike threshold and d*V*/d*t*. It is shown that the activation of hyperpolarizing current preceding spike threshold could prevent inward Na^+^ current from inducing upstroke, which results in a prominent spike threshold dynamics. All these dynamical and biophysical studies could help us to further interpret the mechanism of neural coding.

## Results

### Spike threshold dependence on dV/dt for Type I and Type II neurons

We first adopt a two-dimensional biophysical model (see Materials and methods) to quantitatively investigate the spike threshold for Type I and Type II neurons. Previous studies [4–10, 12–16, 20] have shown that neuronal spike threshold is tightly related to the rate of membrane depolarization preceding a spike (d*V*/d*t*). Thus, we explore the spike threshold in the case of different values of d*V*/d*t*, which is achieved by injecting a cluster of current ramps with different slopes into the neuron model (see Materials and methods). In our study, d*V*/d*t* is defined as the membrane potential change from the time of current ramp onset to offset [20].

**Fig 1:**
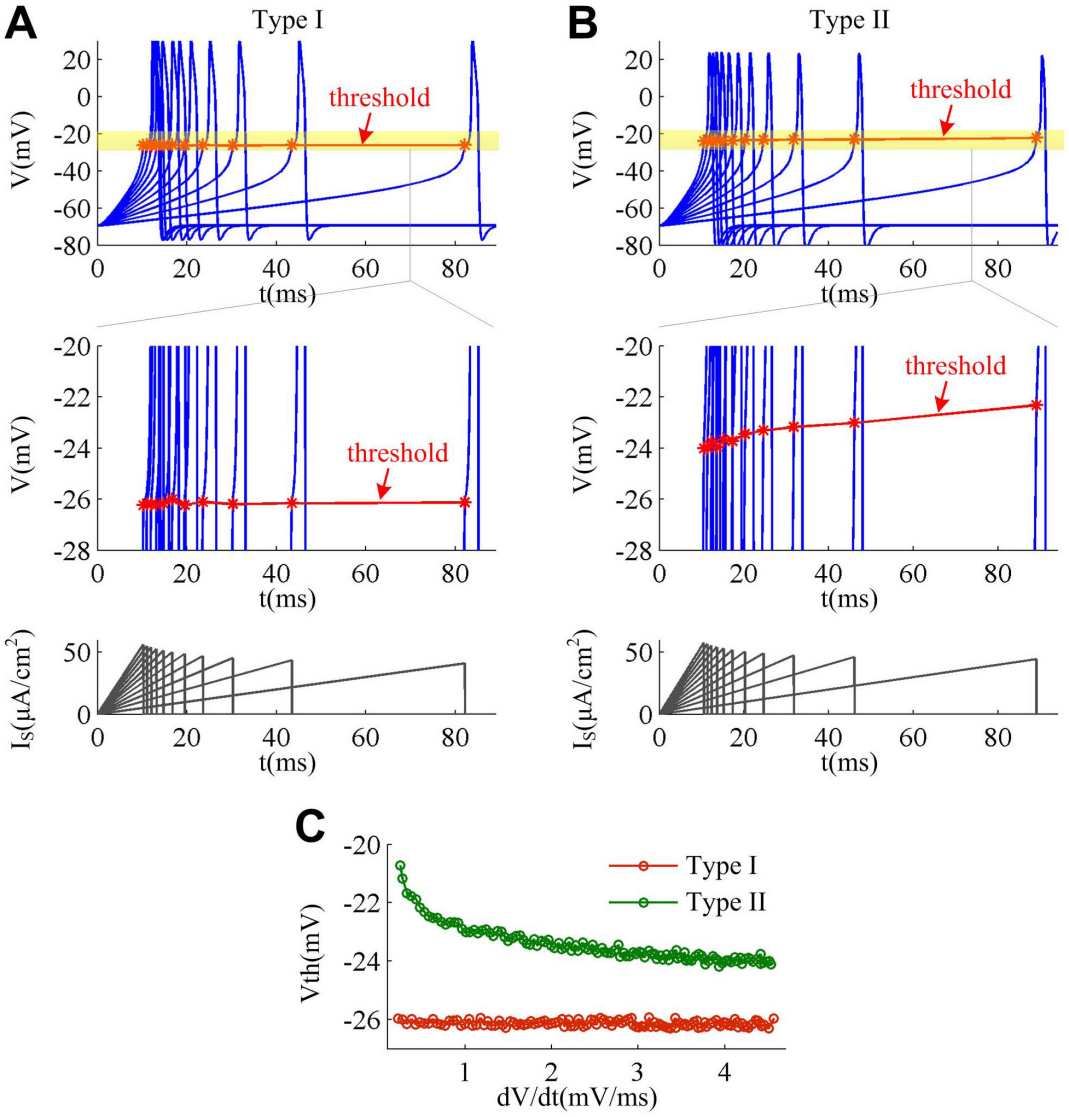
Spike threshold dynamics in two cell types. A set of sample spikes from Type I and Type II neuron as d*V*/d*t* increases from 0.2mV/ms to 4.5mV/ms are respectively shown in (A) and (B). The slope *K* of the corresponding current ramp *I*_s_ is from 0.5μA/(cm^2^·ms) to 5.5μA/(cm^2^·ms) with a step of 0.5μA/(cm^2^·ms). The center panels in (A) and (B) are the enlarge views near the spike threshold (yellow region). (C) gives the spike threshold as a function of d*V*/d*t* for two cell types. “*V*th” denotes the spike threshold.

The relationships between spike threshold and d*V*/d*t* for Type I and Type II neurons derived from the two-dimensional model are shown in Fig 1. It can be observed that the spike thresholds for two types of neuron are distinctly different. In Type I neuron, the spike threshold is insensitive to d*V*/d*t*, which exhibits a very small change between -26.30mV and -25.93mV as d*V*/d*t* is increased from 0.2mV/ms to 4.5mV/ms. That is, Type I model is unable to produce a relationship between spike threshold and d*V*/d*t* (Fig 1C). The range of d*V*/d*t* in our study is consistent with previous modeling [20] and experimental [8] researches. Compared to Type I neuron, Type II neuron always has a more depolarized spike threshold in the observed range of d*V*/d*t*, and its threshold is sensitive to d*V*/d*t* which shows a larger variation between -24.18mV and -20.72mV. Then, Type II neuron could produce a pronounced inverse relation between spike threshold and d*V*/d*t* (Fig 1C), which is different from Type I.

### Dynamical basis of Type I and Type II spike threshold dynamics

With the two-dimensional model, we have determined the spike threshold dependence on d*V*/d*t* for Type I and Type II neurons. Our next step is to use this minimal model to explore the corresponding dynamical basis of the spike threshold associated with each type of neuron model. Since there are two variables in the minimal model, i.e., membrane potential *V* and K^+^ gating variable *w*, we could describe the interactions between them on a phase plane and create a phase portrait by plotting one variable against the other. The phase portrait contains almost all of the qualitative information about neuronal dynamics, and could qualitatively capture them in a physiological way [2, 29, 33]. There is an especially important line on phase plane, i.e., nullcline, which represents the states where neuronal variable remains constant [2, 29]. How the nullclines of different variables interact could help decipher neuronal dynamics and further unfold their complex dynamical behaviors [2, 29, 33].

**Fig 2:**
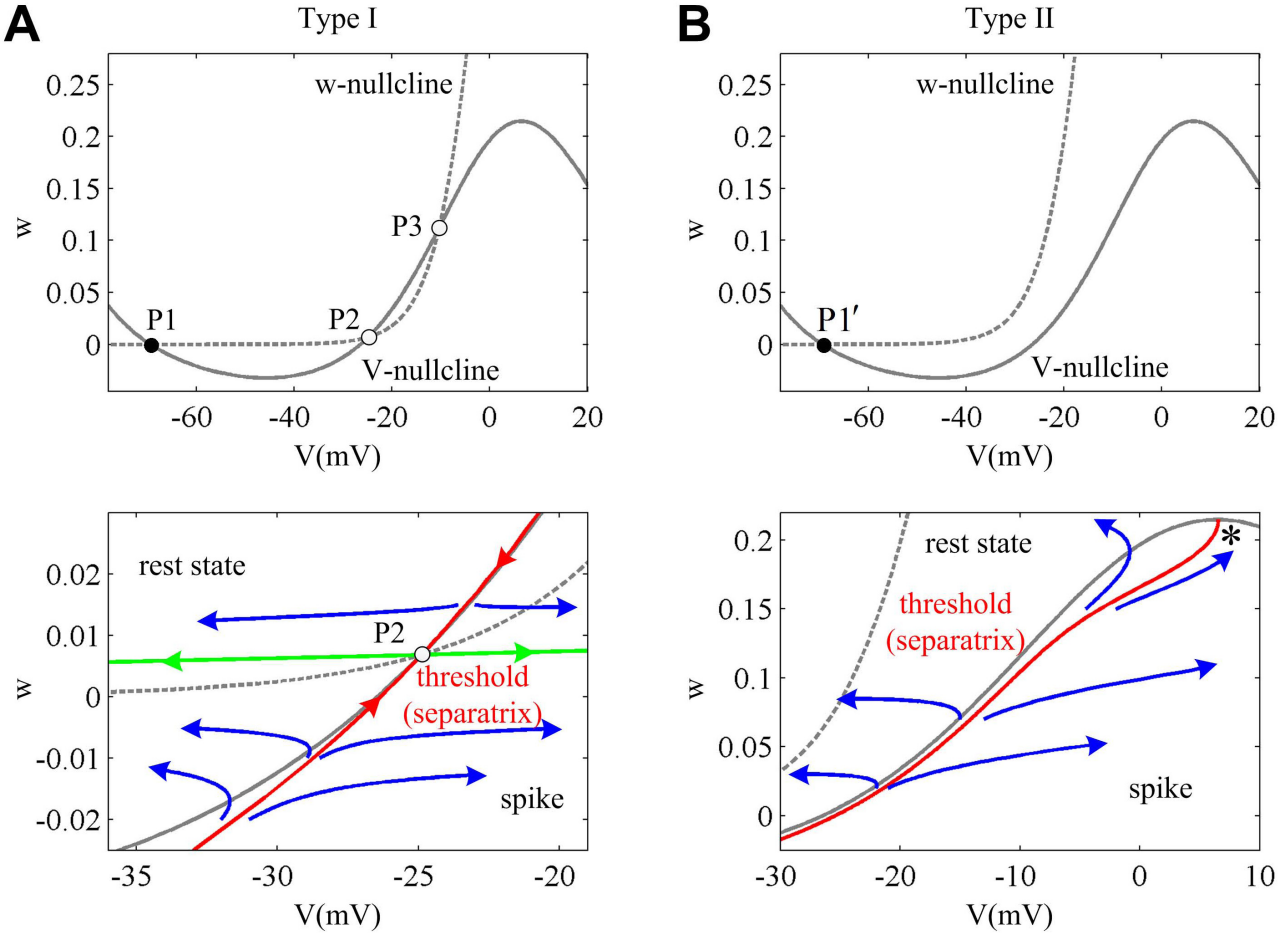
Spike threshold in phase space for two cell types. (A) Type I excitability is generated through a SNIC bifurcation. Before stimulation, there are three intersections between *V*- and *w*-nullclines. P1 is a stable node, P2 is a saddle and P3 is an unstable focus. The stable manifold of saddle P2 (red solid line) is the threshold for Type I neuron, which is also referred as a separatrix since it separates the phase space into two regions with different qualitative behaviors. If membrane potential *V* is not beyond the separatrix, it follows a direct subthreshold route to converge to the stable node P1 which results in spike failure. If *V* exceeds separatrix, its trajectory makes a long excursion to generate a spike and then converges to P1. The green solid lines are two special orbits that start from saddle P2 and end at the stable node P1. The difference between them is that the left orbit is subthreshold while the right one is suprathreshold. (B) Type II excitability is generated through a Hopf bifurcation. Before stimulation, there is only one stable focus P1′ between *V*- and *w*-nullclines. Its threshold that separates suprathreshold response from subthreshold is a special canard trajectory, which is computed by integrating the system backward with a -0.01ms time step from the right vertex of *V*-nullcline (point *).

The phase portraits for two types of neuron model in the absence of current ramp are shown in Fig 2 (top panels). In this case, the *V*- and *w*-nullclines for Type I neuron intersect at three points: a stable node P1, a saddle P2 and an unstable focus P3, which corresponds to a saddle-node bifurcation. Since Type II dynamics is generated through a Hopf bifurcation, the *V*- and *w*-nullclines intersect at only one stable focus P1′ without external stimulus. The stable intersection (i.e., system equilibrium) could attract *V* trajectories, which is also called an attractor. However, depending on neuronal prior state, there are two different paths that *V* trajectory moves to the stable equilibrium. One is that *V* trajectory follows a more direct subthreshold route and generates a subthreshold response, the other is the trajectory makes a long excursion to generate an action potential and finally converges to the attractor. The manifold separating these two paths is called separatrix first described by Fitzhugh [34, 35], because it separates the phase space into two regions that have different qualitative behaviors. If membrane potential *V* is not beyond the separatrix, it is subthreshold and results in spike failure, while those crossing it are suprathreshold and could produce spikes (bottom panels, Fig 2). Thus, the separatrix is also referred as the spike threshold [2, 29, 32, 34, 35]. We have plotted the threshold curves for two cell types, which are shown in the bottom panels of Fig 2. It can be seen that their separatrix is different from each other. For Type I neuron, the stable manifold of saddle P2 (red solid line) is its threshold. For Type II neuron, its threshold is a special canard trajectory, which follows the unstable branch of its *V*-nullcline all the way and moves to the right knee point (*). Fitzhugh [34, 35] refers to this as a quasi-threshold. The difference in separatrix between two cell types could result in different spike threshold dependence on d*V*/d*t*, which will be elaborated in the following part.

**Fig 3:**
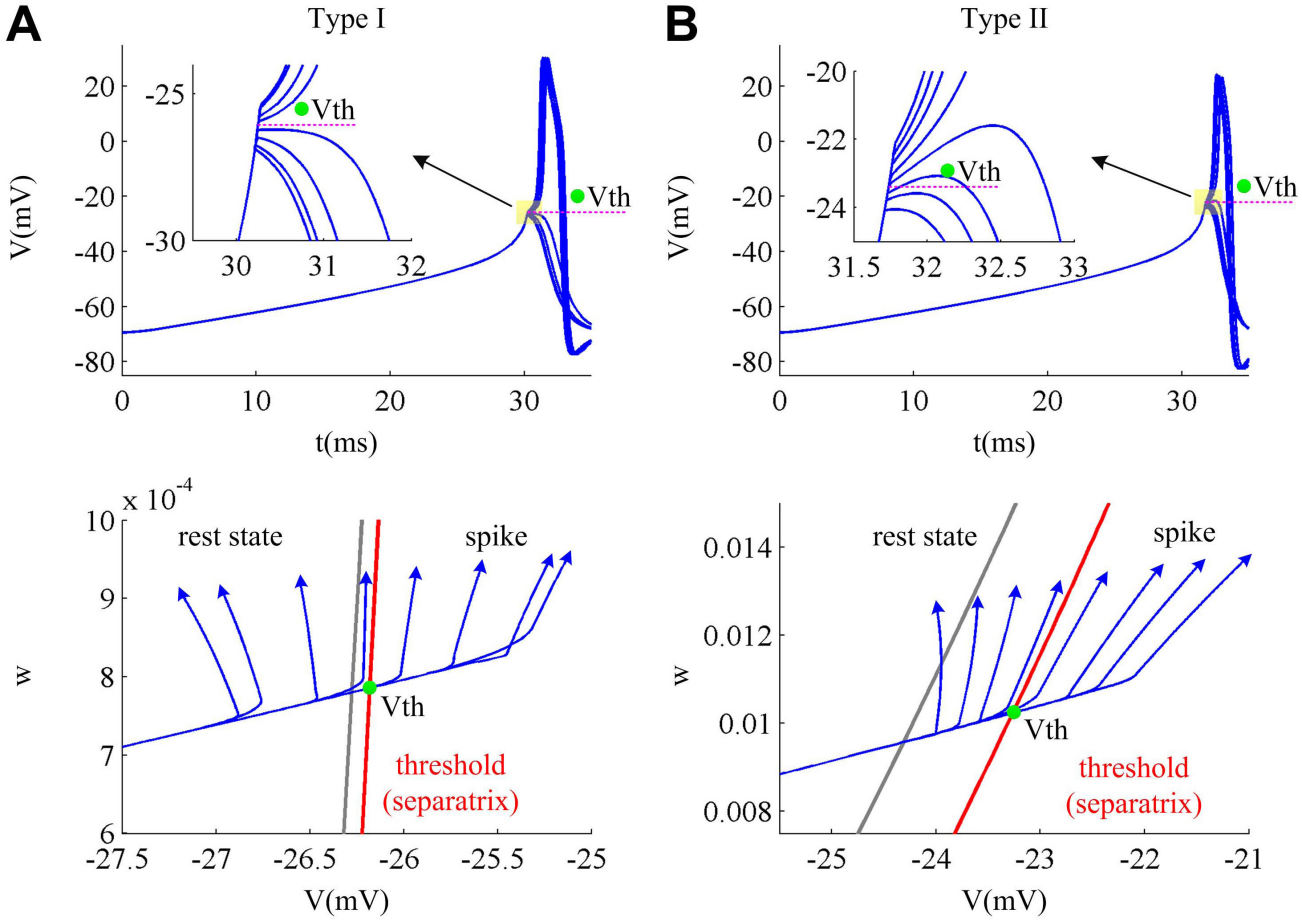
Phase plane geometry of the method to determine spike threshold. The responses and relevant phase plane geometries for Type I and Type II neurons to a set of current ramps are respectively shown in (A) and (B). When ramp slope *K* is fixed, i.e., *K* = 1.5μA/(cm^2^·ms), increasing ramp duration can drive *V* trajectory to move towards the separatrix (red solid line) at the rate of d*V*/d*t*. If *I*_S_ is sufficient to drive *V* trajectory across separatrix, there is a spike generated when *I*_S_ is removed. If *I*_S_ is not high enough to drive *V* trajectory beyond separatrix, the spike cannot be initiated after removing *I*_S_. The green dots represent the spike threshold, and the gray solid line is the *V*-nullcline.

For the current ramp with fixed slope, the membrane potentials of two cell types both increase as the stimulus duration extends, as shown in Fig 3. If the ramp duration is not high enough to make membrane potential *V* reach spike threshold curve, the *V* trajectory will converge to the stable equilibrium along a direct subthreshold route after removing the stimulus, which results in spike failure. When the ramp duration is sufficient to drive *V* beyond spike threshold curve, the neuron could generate a spike after removing the stimulus (bottom panels, Fig 3).

**Fig 4:**
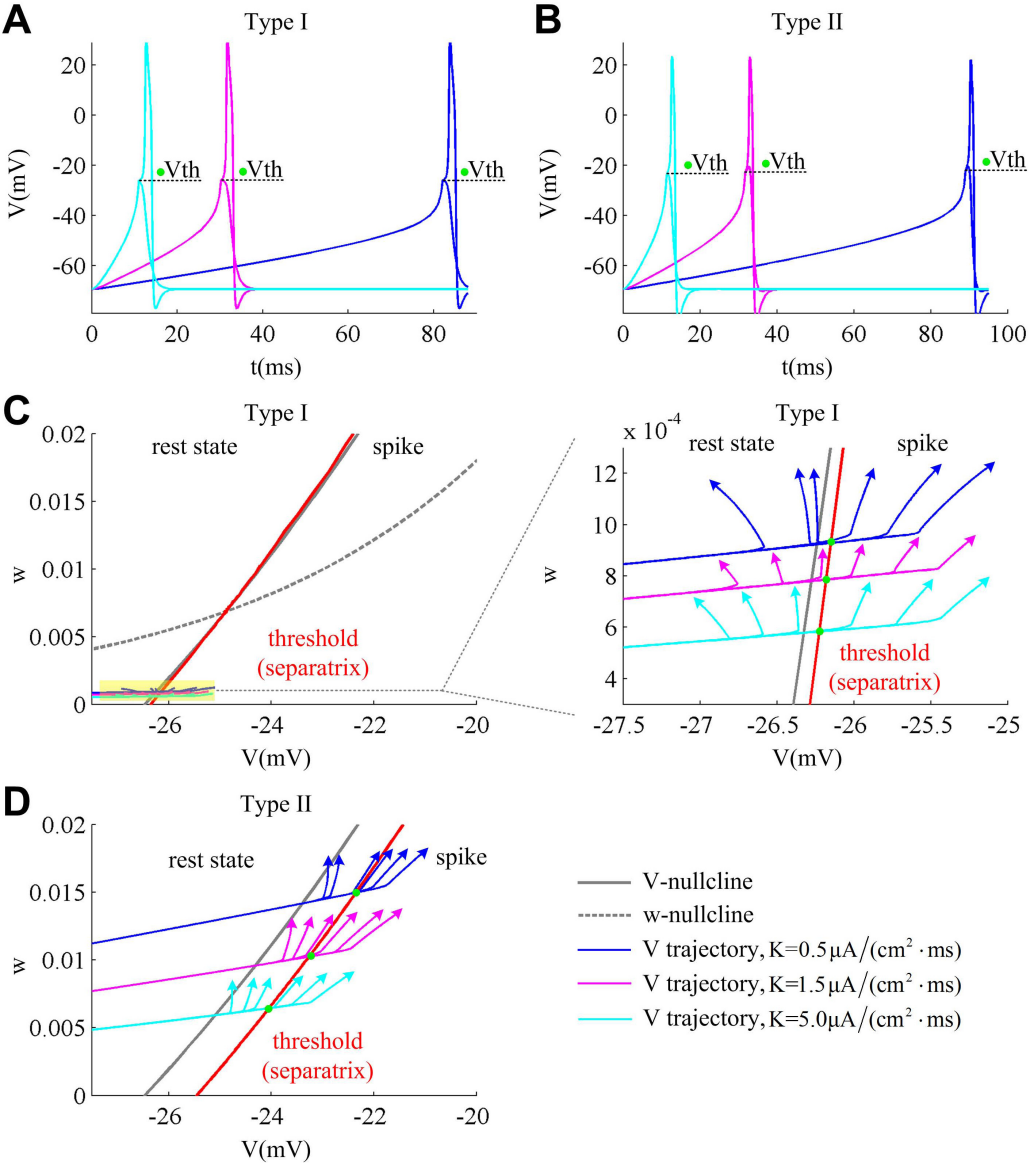
Dynamical explanation of the spike threshold dependence on d*V*/d*t* associated with each cell type. (A) and (B) respectively give the sample subthreshold and suprathreshold responses of each cell type to current ramp with *K* = 0.5μA/(cm^2^·ms) (blue), 1.5μA/(cm^2^·ms) (pink) and 5.0μA/(cm^2^·ms) (cyan blue). (C) and (D) respectively describe the separatrix (i.e., threshold) of two types of excitability on phase plane. The right panel in (C) is the enlarged view of the yellow region in its left panel. The separatrix of Type I neuron lies on the left side of Type II, which corresponds to a hyperpolarized spike threshold. As ramp slope *K* changes, the variable *w* in Type II neuron varies in a much larger scale compared to Type I. Then, varying d*V*/d*t* could produce an obvious change in the spike threshold of Type II neuron. The green dots denotes the spike threshold *V*th.

**Fig 5:**
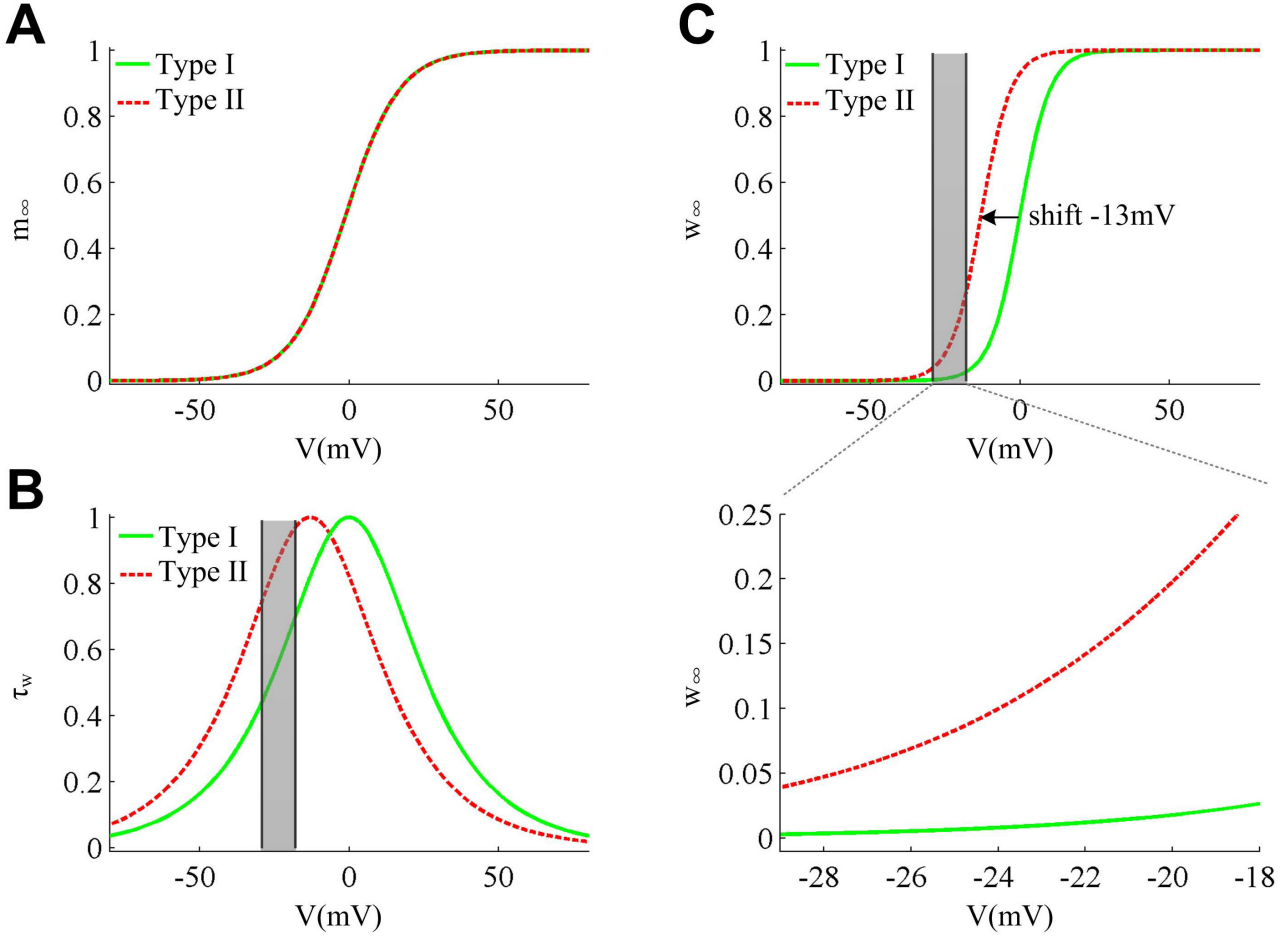
Activation curves and kinetics of ionic currents in two cell types. (A) The steady-state Na^+^ activation *m*_∞_ is the same for Type I (green solid line) and Type II (red dotted line) neurons. (B) Voltage-dependent time constant *τ*_*w*_ for two cell types. At the perithreshold potentials (gray region), Type II neuron has a larger *τ*_*w*_ than Type I, which indicates its variable *w* has a slower kinetics than Type I. (C) The half-activation potential of *w*_∞_ in Type II neuron is hyperpolarized by -13mV relative to Type I. Then, its *w*_∞_ is much larger at the perithreshold potentials than Type I, which corresponds to a larger scale variation of the variable *w* in Type II neuron (Fig 4D).

The slope of the current ramp controls the rate of membrane depolarization preceding a spike, i.e., d*V*/d*t*. With larger ramp slope *K*, the membrane potential *V* of two cell types both rises at a higher speed, which leads to a larger value of d*V*/d*t* (Fig 4A and 4B). In the same way, the lower ramp slope *K* leads to a smaller value of d*V*/d*t*. Different d*V*/d*t* results in the variation of spike threshold. However, the relationship between spike threshold and d*V*/d*t* is different for Type I and Type II neurons. To compare and understand the difference in the spike threshold of two types of neuron model, we plot their separatrix on the same coordinate axes (Fig 4C and 4D). It can be observed that the Type I separatrix lies at the left side of Type II on the phase plane, which indicates that its spike threshold is more hyperpolarized than Type II neuron as a whole. For both cell types, their variable *w* is higher when d*V*/d*t* is lower. This is because the slower ramp that corresponds to lower value of d*V*/d*t* is more prone to activate slower gating variable *w* and makes it reach a higher value at the subthresholds. Then, the spike threshold moves to the lower potential along the threshold as d*V*/d*t* increases. Moreover, as ramp slope *K* increases from 0.5μA/(cm^2^·ms) to 5μA/(cm^2^·ms), the Type II *V* trajectory shows a larger scale variation (about ten times) along the *w*-axis relative to Type I (Fig 4C and 4D). This is due to their different activation curves and kinetics of K^+^ current. We found that although the Na^+^ activation occurs at the same potential for two cell types (Fig 5A), the K^+^ channel dynamics are different. Compared to Type I, the K^+^ time constant *τ*_*w*_ is bigger in Type II neuron at the perithreshold potentials (Fig 5B), which indicates that the kinetics of its variable *w* are slower. Then, the same slow ramp is more prone to activate Type II variable *w* than Type I. Meanwhile, the half-activation potential of K^+^ steady-state activation curve *w*_∞_ for Type II neuron is hyperpolarized by -13mv (Fig 5C) relative to Type I, which indicates its K^+^ activation occurs at a more hyperpolarized potential. This leads to much larger values of *w*_∞_ at the perithreshold potentials for Type II neuron model (bottom panel, Fig 5C). Therefore, its gating variable *w* could vary in a much larger range around the threshold potentials than Type I.

These stimulations bring the following conclusions. First, the Type II spike threshold curve lies at the right side of Type I, which results in a more depolarized threshold. Second, the slower ramp leads to a lower d*V*/d*t* and higher *w*, which makes spike threshold get smaller as d*V*/d*t* increases. Third, the Type II *V* trajectories vary in a much larger scale along the *w*-axis than Type I in the case of the same ramp slopes. Therefore, as d*V*/d*t* increases, the Type II spike threshold could have a relatively obvious change while Type I threshold shows little difference. Then, there is a pronounced relationship between spike threshold and d*V*/d*t* in Type II neuron, which is missing in Type I.

### Biophysical explanation of the Type I and Type II spike threshold dynamics

With phase plane analysis of the two-dimensional model, we have identified the dynamical basis of the spike threshold dependence on d*V*/d*t* for each cell type. In fact, spike generation is also a phenomenon that results from the nonlinear interactions between opposite, kinetically mismatched membrane currents [29, 36–39], which could not only shape spike waveform at suprathreshold potentials but also control spike initiation at the perithreshold potentials. Specifically, the inward currents (i.e., Na^+^ current) mainly depolarize membrane potential and produce the rapid upstroke of spike by positive feedback (i.e., self-sustaining), while the outward currents (i.e., K^+^ and leak current) mainly hyperpolarize membrane potential and result in the downstroke of spike through negative feedback [2, 29, 38–40]. Here, we investigate how the interactions between two opposite currents at the threshold potentials contribute to the spike threshold dynamics of two types of neuron model.

**Fig 6:**
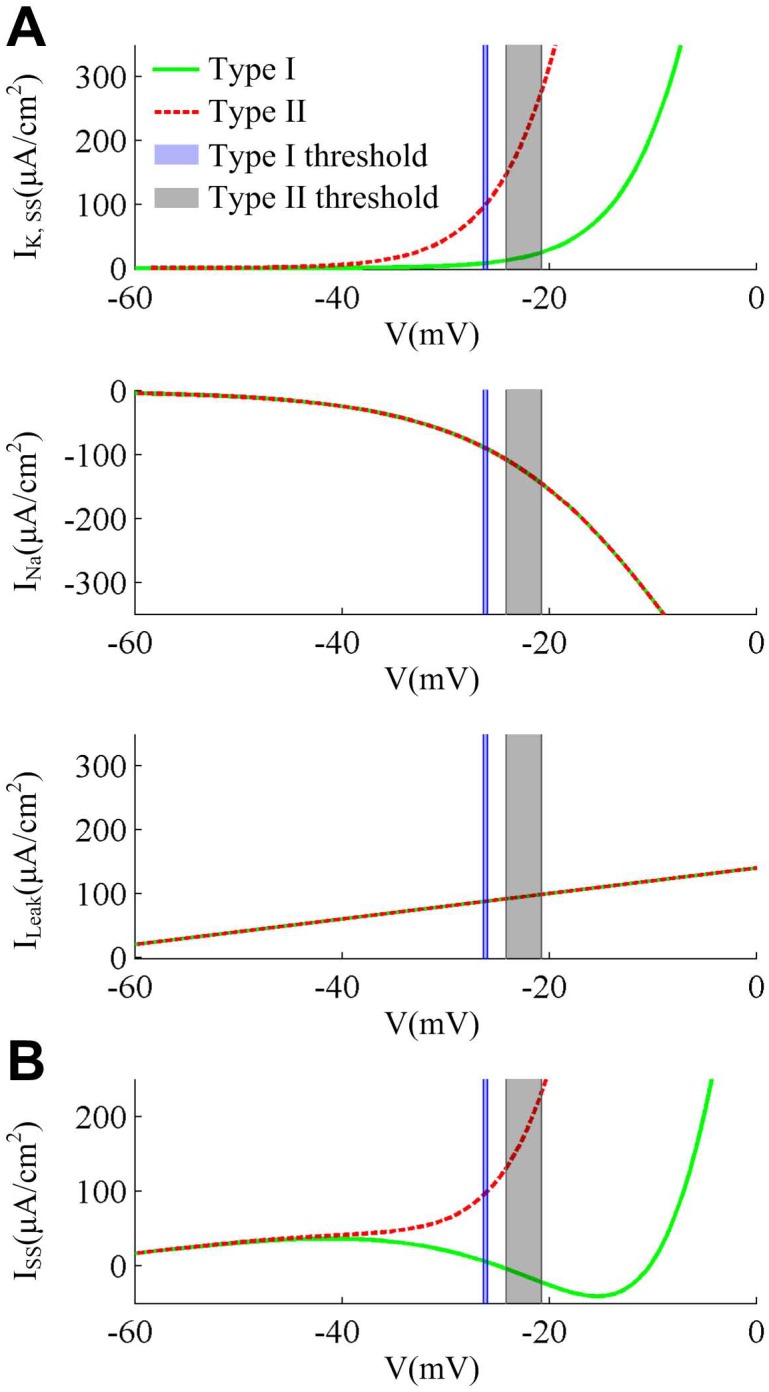
Steady-state membrane currents at the subthresholds for two cell types. (A) The individual steady-state membrane currents at the subthreshold potentials for each cell type. Type II steady-state K^+^ current (*I*_K,SS_) has activated prior to spike threshold (gray region), whereas this current does not activate at the threshold potentials (blue region) in Type I neuron. The other two currents, i.e., Na^+^ current (*I*_Na_) and leak current (*I*_Leak_), have the same subthreshold property for two cell types. (B) The steady-state total membrane current *I*_SS_ at the subthreshold potentials, i.e., *I*_SS_-*V* curve. The Type II *I*_SS_ is outward at the perithresholds which arises from the activation of outward K^+^ current prior to threshold. In contrary, the Type I *I*_SS_ is inward which is much smaller than Type II. The total current *I*_SS_ is computed as the sum of all individual currents, i.e., K^+^, Na^+^ and leak currents.

Results in Fig 5 show that the K^+^ half-activation potential for Type II neuron is hyperpolarized by -13mV relative to Type I, which results in the different subthreshold properties of *I*_K_ for two cell types (top panel, Fig 6A). The outward *I*_K_ in Type I neuron activates at higher potentials than inward *I*_Na_, while it activates at roughly the same potential as *I*_Na_ in Type II neuron. The different activations of outward *I*_K_ relative to inward *I*_Na_ lead to different shapes of steady-state membrane current *I*_SS_-potential *V* (i.e., *I*_SS_-*V*) curve (Fig 6B). For Type I neuron, the *I*_SS_-*V* curve is non-monotonic and has a region of negative slope prior to threshold, whereas it is monotonic for Type II. Then, the Type II net current *I*_SS_ is outward at the threshold potentials, which is much larger than that of Type I neuron (Fig 6B). To generate a spike, the inward *I*_Na_ in Type II neuron must activate more rapidly to outrun outward *I*_K_ to become self-sustaining and produce the suprathreshold depolarization. However, the competition between two opposite currents is missing in Type I cell, since its outward *I*_K_ has not yet activated when inward *I*_Na_ drives membrane potential *V* through threshold to initiate a spike. Namely, the presence of a more net outward current *I*_SS_ prior to spike initiation could drive potential *V* to reach a more depolarized value before inward *I*_Na_ becomes self-sustaining. Then, Type II neuron generates a more depolarized spike threshold than Type I.

**Fig 7:**
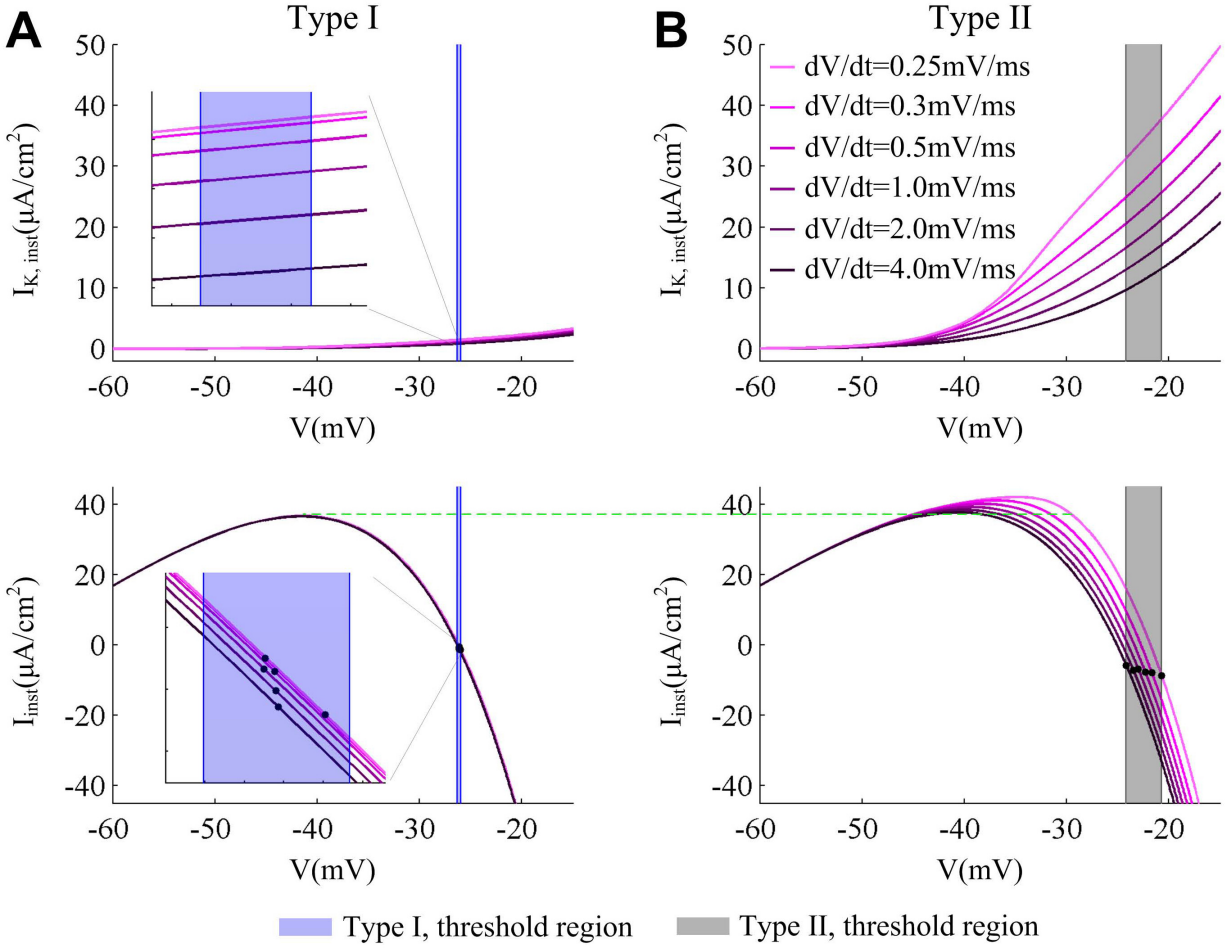
Instantaneous K^+^ and net currents at the subthresholds for two cell types. (A) and (B) respectively show the instantaneous K^+^ current (*I*_K,inst_) and net current (*I*_inst_) for two types of neurons in the case of several d*V*/d*t*. Black dots stand the value of *I*_inst_ at the spike threshold. As d*V*/d*t* decreases, the outward *I*_K,inst_ becomes more prominent in Type II neuron, which leads to more net outward current *I*_inst_ prior to spike threshold. Unlike Type II, the *I*_K,inst_ in Type I neuron has not activated at the threshold potentials (blue region). As d*V*/d*t* changes, there are only extremely small changes in its *I*_K,inst_ and *I*_inst_.

Further, under slow current ramp stimulus, the instantaneous outward *I*_K_ in Type II neuron progressively becomes stronger around its threshold potentials as d*V*/d*t* decreases, which leads to a more prominent instantaneous net outward current *I*_inst_ (right panels, Fig 7). Since there is more outward current antagonizes inward Na^+^ prior to spike threshold, the membrane potential *V* has to reach a more depolarized value before inward *I*_Na_ becomes self-sustaining, which results in a more depolarized spike threshold. Thus, Type II neuron has a pronounced relationship between spike threshold and d*V*/d*t*. Unlike Type II, the outward *I*_K_ in Type I neuron has very small variations at its threshold potentials with different values of d*V*/d*t*, since it has not yet activated at these potentials (left panels, Fig 7). Then, its instantaneous net current *I*_inst_ could only vary in a very narrow range with different slower ramps (Fig 7A), which leads to a tight clustering of spike threshold. Thus, there is no dynamic spike threshold appearing with increasing d*V*/d*t* in Type I neuron. In other word, the activation of outward current prior to spike initiation could lead to a stronger net outward current at the perithreshold potentials, which produces a more depolarized spike threshold as well as an inverse relation between spike threshold and d*V*/d*t*.

### Effects of varying other model parameters on spike threshold dynamic

In our previous stimulation, we have found different spike threshold dynamics by increasing d*V*/d*t* in two types of neuron model. Through analyzing the subthreshold properties of membrane currents, it is shown that Type II neuron has a much larger outward current prior to spike initiation than Type I, which produce a relationship between spike threshold and d*V*/d*t*. Here, we further test whether the spike threshold dependence on d*V*/d*t* could be inferred from two types of excitability. To reproduce Type I and Type II excitability, we start our research by varying β_*w*_ in the minimal model. In fact, varying other parameters that control K^+^ or Na^+^ channels, such as γ_*w*_, ${\bar{\text{g}}}_{\text{K}}$, β_*m*_ or ${\bar{g}}_{\text{Na}}$, could also affect spike initiation dynamics and generate two types of excitability [29]. Therefore, we respectively examine whether our previous prediction and biophysical explanation are supported in the case of varying these four parameters.

**Fig 8:**
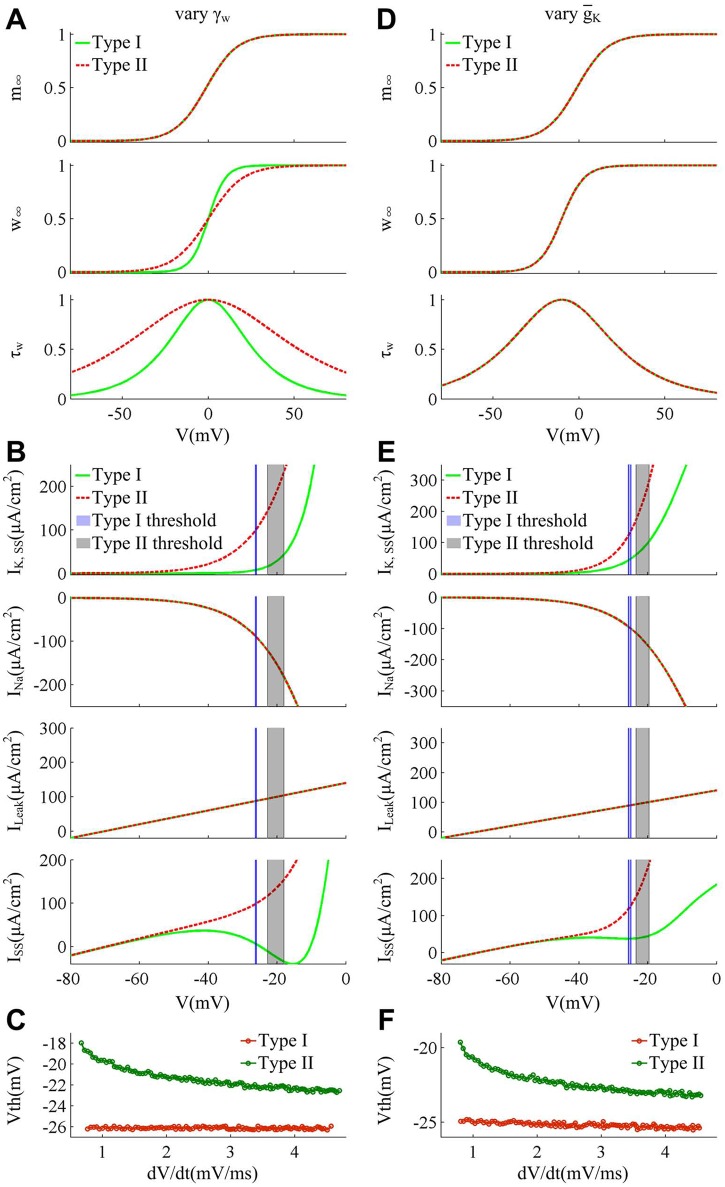
Spike threshold and corresponding biophysical basis produced by varying γ_*w*_ and ${\bar{\text{g}}}_{\text{K}}$. (A)-(C) summarize the results in the case of varying γ_*w*_. The minimal model exhibit Type I excitability with γ_*w*_ = 10mV and Type II excitability with γ_*w*_ = 20mV. (D)-(F) summarize the results in the case of varying ${\bar{\text{g}}}_{\text{K}}$. The neuron exhibits Type I excitability with ${\bar{\text{g}}}_{\text{K}}=7{\text{mS}/\text{cm}}^{2}$ and Type II excitability with ${\bar{\text{g}}}_{\text{K}}=20{\text{mS}/\text{cm}}^{2}$. β_*w*_ = 0mV for (A)-(C), and β_*w*_ = -10mV, γ_*w*_ = 13mV for (D)-(F). Other parameters are as indicated in Materials and Methods. (A) and (D) show the activation curves and kinetics of ionic currents for two cell types. Increasing γ_*w*_ has no effects on Na^+^ activation *m*_∞_, while it reduces the slope of K^+^ activation *w*_∞_ and increases K^+^ time constant *τ*_*w*_. Unlike γ_*w*_, increasing ${\bar{\text{g}}}_{\text{K}}$ has no impacts on the voltage-dependency and kinetics of *I*_K_, while it affects the magnitude of K^+^ conductance (data is not shown). (B) and (E) give the membrane currents at the subthresholds for two types of neuron model. Increasing γ_*w*_ has no effects on *I*_Na_ and *I*_Leak_, whereas it leads *I*_K,SS_ to activate prior to spike initiation. Increasing ${\bar{\text{g}}}_{\text{K}}$ causes the outward *I*_K,SS_ to be much stronger without altering its voltage-dependency and kinetics. Both of them result in a much larger outward *I*_SS_ at the threshold. (C) and (F) give the spike threshold within a range of d*V*/d*t* for each cell. The d*V*/d*t* is from 0.8mV/ms to 4.5mV/ms. This is because Type II neuron generates the delayed loss of stability for d*V*/d*t*<0.8mV/ms, which leads to a big error when computing d*V*/d*t*. The specific reason is shown in the Discussion.

Increasing γ_*w*_ and ${\bar{\text{g}}}_{\text{K}}$ could both boost the K^+^ current intensity at the subthreshold potentials and make the neuron convert from Type I to Type II excitability [29]. We summarize the spike threshold and relevant biophysical explanation for these two cases in Fig 8. The specific biophysical mechanism for each case is different. Increasing γ_*w*_ reduces the slope of *w*_∞_ and increases time constant *τ*_*w*_ (Fig 8A), which causes *I*_K_ to be more strongly activated by perithreshold depolarization (top panel, Fig 8B). In contrary, increasing ${\bar{\text{g}}}_{\text{K}}$ cannot alter the voltage-dependency or subthreshold kinetics of *I*_K_ (i.e., *w*_∞_ and *τ*_*w*_), while it increases the conductance magnitude of K^+^ current, i.e., *g*_K_, which has a comparable effect on *I*_K_ with that produced by increasing γ_*w*_ (Fig 8E). Both of them could lead to a more prominent net outward current at the threshold potentials (bottom panels, Fig 8B and 8E). By quantifying the spike thresholds with increasing d*V*/d*t* in both cases, it is found that Type II neuron indeed has a more depolarized spike threshold as well as an inverse relation between spike threshold and d*V*/d*t* relative to Type I (Fig 8C and 8F), which is in accordance with the prediction of varying β_*w*_.

**Fig 9:**
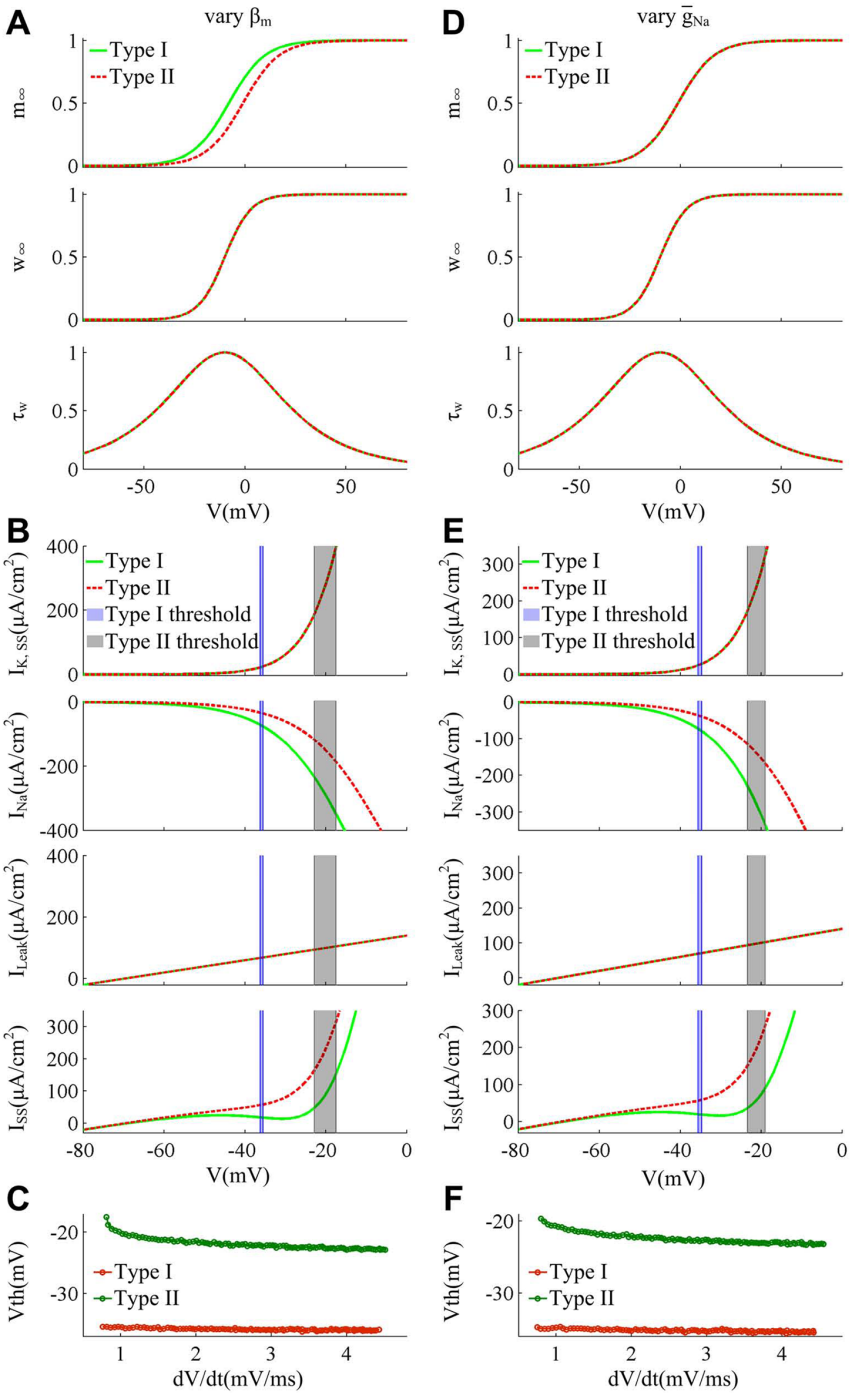
Spike threshold and corresponding biophysical basis produced by varying β_m_ and ${\bar{\text{g}}}_{\text{Na}}$. (A)-(C) summarize the results produced by varying β_m_. The minimal model exhibits Type I excitability with β_m_ = -8mV and Type II excitability with β_m_ = -1mV. (D)-(F) summarize the results produced by varying ${\bar{\text{g}}}_{\text{Na}}$. The model exhibit Type I excitability with ${\bar{\text{g}}}_{\text{Na}}=40{\text{mS}/\text{cm}}^{2}$ and Type II excitability with ${\bar{\text{g}}}_{\text{Na}}=20{\text{mS}/\text{cm}}^{2}$. (A) and (D) show the activation curves and kinetics of ionic currents for two cell types. Increasing β_m_ depolarizes Na^+^ activation *m*_∞_ and produces no effects on *w*_∞_ as well as *τ*_*w*_. Decreasing ${\bar{\text{g}}}_{\text{Na}}$ reduces the magnitude of Na^+^ conductance (data is not shown) without altering *m*_∞_, *w*_∞_ and *τ*_*w*_. (B) and (E) give the membrane currents at the subthresholds for two types of neuron. Depolarizing Na^+^ activation *m*_∞_ causes *I*_Na_ to be less strongly activated at the perithreshold potentials. Decreasing ${\bar{\text{g}}}_{\text{Na}}$ reduces the *I*_Na_ intensity without altering its voltage-dependency and kinetics. Both of them result in a much larger outward *I*_SS_ at the threshold. (C) and (F) show the spike threshold as a function of d*V*/d*t* for two cell types. The d*V*/d*t* range is from 0.8mV/ms to 4.5mV/ms. For both cases, β_*w*_ = -10mV, γ_*w*_ = 13mv, and other parameters are as indicated in Materials and Methods.

Varying β_*m*_ and ${\bar{\text{g}}}_{\text{Na}}$ could affect the intensity of inward *I*_Na_ at the subthreshold potentials and switch excitability between Type I and Type II by different biophysical mechanisms [29]. Increasing β_*m*_ shifts *m*_∞_ to a depolarized potential (Fig 9A), which makes the inward *I*_Na_ less strongly activated at the perithreshold potentials (Fig 9B). Decreasing ${\bar{\text{g}}}_{\text{Na}}$ without altering *m*_∞_ weakens the conductance magnitude of Na^+^ current, i.e., *g*_Na_, which has the comparable effects with increasing ${\bar{\text{g}}}_{\text{K}}$. In each case, there is both a prominent net outward current at the threshold potentials (bottom panels, Fig 9B and 9E), which leads to a depolarized spike threshold as well as an inverse relation between threshold and d*V*/d*t* (Fig 9C and 9F). Thus, a consistent result is also obtained in these two cases with that of varying β_*w*_.

**Fig 10:**
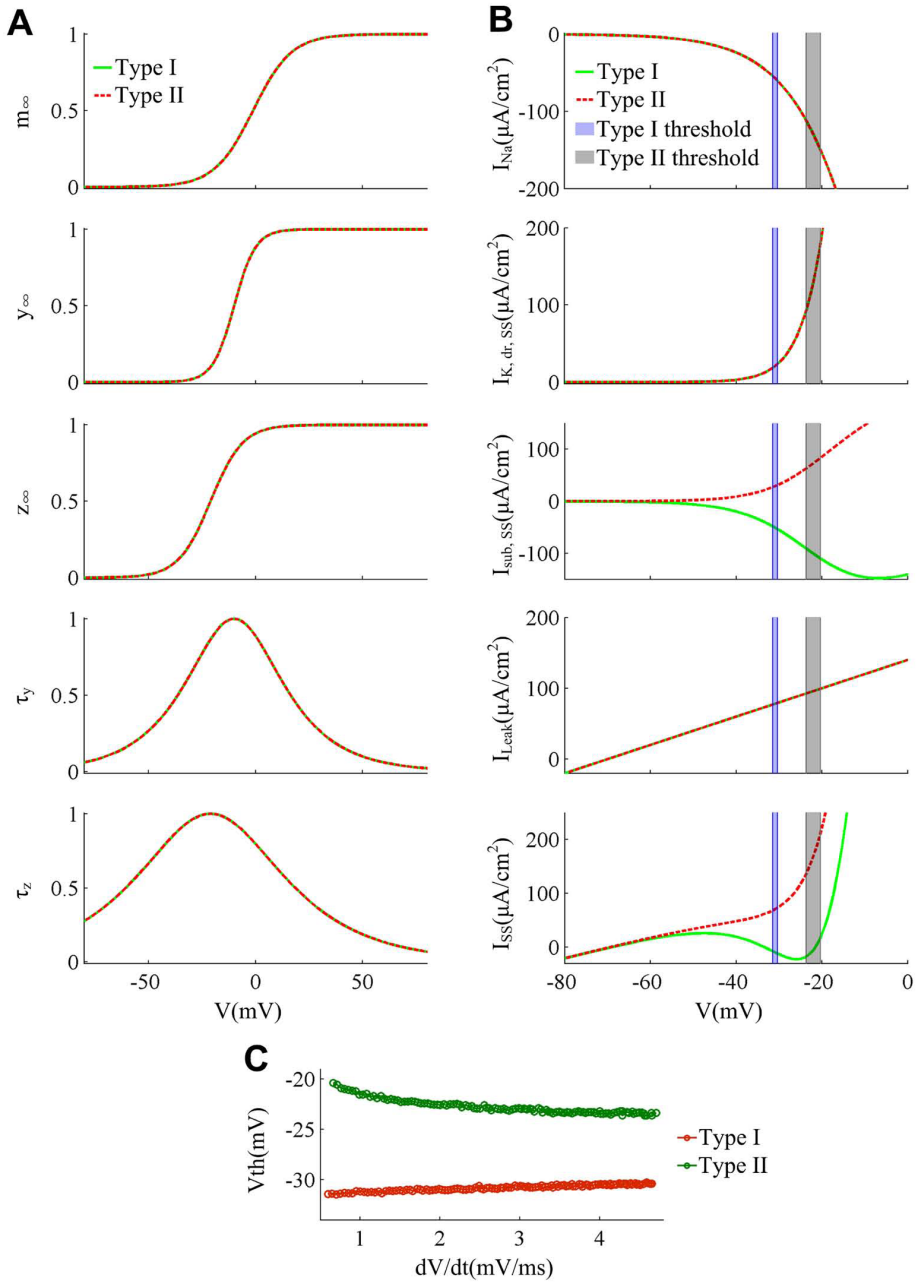
Spike threshold and corresponding biophysical basis produced by realistic model. (A) Activation curves and kinetics of ionic currents for each cell type. There are three ionic currents, i.e., *I*_Na_, *I*_Sub_ and *I*_K,dr_, in three-dimensional model. The steady-state activations and voltage-dependent time constants of these currents are all the same for Type I (green solid line) and Type II (red dotted line) neurons. (B) Membrane currents at the subthreshold potentials. For two cell types, the subthreshold properties of *I*_Na_, steady-state *I*_K,dr_ (i.e., *I*_K,dr,SS_) and *I*_Leak_ are identical, while the direction and magnitude of *I*_Sub_ is different. It is inward in Type I neuron and outward in Type II neuron, which makes Type II neuron have a much larger outward *I*_SS_ at the threshold potentials. (C) Spike threshold within a range of d*V*/d*t* for each cell type. The d*V*/d*t* range is from 0.6mV/ms to 4.5mV/ms, since there is the delayed loss of stability appearing in Type II neuron with. d*V*/d*t*<0.6mV/ms.

Finally, we adopt a three-dimensional model to further test our prediction and biophysical explanation (Fig 10). The complex neuron is obtained by splitting outward *I*_K_ into *I*_K,dr_ and *I*_Sub_ [29], which could increase the biophysical realism of the model and make it arguably better for biophysical interpretation. With fixing the voltage-dependent of *I*_K,dr_ and *I*_Na_, the neuron can be converted between two cell types by varying the magnitude and direction of *I*_Sub_. When *I*_Sub_ is inward, the neuron exhibit Type I excitability, and outward for Type II excitability [29]. Then, relative to Type I, a much larger net outward current appears at the threshold potentials in Type II neuron (bottom panel, Fig 10B). By determining the spike threshold in a range of d*V*/d*t*, it is observed that Type II neuron indeed has a more depolarized spike threshold and a robust inverse relation between spike threshold and d*V*/d*t* compared with Type I (Fig 10C). Therefore, our prediction and biophysical explanation are also reproducible in more biophysically realistic model.

## Discussion

The spike threshold in many types of neuron has been shown to be dependent on the rate of the membrane depolarization, i.e., d*V*/d*t*. In this study, we adopt a novel approach [20] to precisely determine the spike threshold of two types of excitability first described by Hodgkin within a range of d*V*/d*t* based on a simple biophysical model. It is shown that the spike threshold in Type II neuron is more depolarized and more sensitive to d*V*/d*t* than that in Type I neuron. With phase plane analysis, we identify the threshold manifold of two cell types and explain how their spike threshold depends on d*V*/d*t*. By characterizing the membrane currents at the threshold potentials, we successfully uncover how the intrinsic properties of inward and outward currents contribute to the spike threshold dynamics associated with each type of excitability.

### Threshold curve in phase space

It has been well established that Type I dynamic is generated through a SNIC bifurcation and Type II is through a Hopf bifurcation [2, 29–32]. The SNIC bifurcation involves a saddle in phase space, while this kind of equilibrium cannot appear in the case of Hopf bifurcation. This leads to the different spike threshold in two cell types. For SNIC bifurcation, the stable manifold of the saddle could be regarded as a separatrix, by which the phase space is divided into two regions with different qualitative behaviors. The small external perturbation cannot drive membrane potential to pass through separatrix, so its solution will decay and there is no spike generated. If the stimulus is sufficient to force membrane potential across separatrix, its solution will grow away exponentially thereby producing a spike. That is, according to the position where the trajectory starts relative to the separatrix, one can accurately predict whether an action potential is initiated. This is commonly referred to as “all-or-none” action potential, and the separatrix is the Type I threshold curve. In this case, the neuron has a well-defined threshold manifold. For Hopf bifurcation, there is no saddle appearing in the phase space, and it does not have the well-defined threshold manifold produced by saddle. The Type II threshold curve is a special canard trajectory which follows the unstable branch of the cubic nullcline to its right knee point. Since the threshold curve and K^+^ current kinetics are different, Type I and Type II neurons generate disparate threshold dynamics.

The current ramp in our study is just a driving force to alter neuronal initial state, which mainly interferes in subthreshold dynamics prior to spike initiation and leads to different rates of membrane depolarization. If the neuron is forced to reach a position just across separatrix, we remove the ramp. Since the *V*-nullcline and separatrix move instantaneously as stimulus changes, the phase portrait of the neuron at the time of ramp offset is the same as that before ramp onset. In this manner, the action potential is purely due to the Na^+^ activation, which has nothing to do with stimulus. The membrane potential at the time of ramp offset corresponds to that just makes inward Na^+^ become self-sustaining, i.e., spike threshold. The ramp slope controls the d*V*/d*t*, and different d*V*/d*t* leads the neuron to stop at different places close to separatrix. Due to the distinct kinetics of K^+^ gating variable *w* in Type I and Type II neurons, the position where their membrane potential stops exhibits disparate evolutions as d*V*/d*t* increases. Then, with this approach, we could account for how spike threshold depends on d*V*/d*t* in two types of neurons according to their phase portraits.

The concept of separatrix is first proposed by Fitzhugh [34, 35], and afterward Izhikevich [2, 32] uses it to decipher the difference of the threshold manifold between Type I and Type II neurons. Both of them mainly consider neuronal responses to brief pulse or to prolonged inhibitory and excitatory steps. Recently, the separatrix is used to interpret the coding properties associated with Type III neurons, which is another Hodgkin’s excitability [2, 29]. For instance, Prescott et al [29] adopt separatrix to explain how Type III neurons generate a single spike to external current steps; Meng et al [41] use separatrix to decipher the mechanism underlying slope sensitivity and coincidence detection of Type III excitability. Although the predictive power of separatrix has been confirmed by these studies, it still does not provide a satisfying dynamical explanation of how spike threshold depends on d*V*/d*t* in Type I and Type II neurons. The present work is the first study to address this problem with phase plane geometry, which enables us to visualize and uncover why two cell types have different spike threshold dependence on d*V*/d*t*. Hopefully, this could lead to an increased application of separatrix in the related fields.

### Roles of outward K^+^ current in threshold dynamic

The method for measuring threshold allows us to investigate the properties of the ionic currents as well as their gating variables at the spike threshold. It is found that the outward K^+^ current activates prior to spike threshold in Type II neuron. Then, it prevents inward Na^+^ current from becoming self-sustaining at a fixed voltage and leads to a more prominent net outward current prior to spike initiation, which results in a depolarized spike threshold and produces an inverse relation between threshold and d*V*/d*t*. In Type I neuron, the outward K^+^ current has not yet activated at the threshold potentials. In this case, it cannot antagonize the inward Na^+^ current, which fails to produce such inverse relation. This suggests that the activation of outward K^+^ current prior to spike initiation is a major factor that regulates the threshold dynamics in two types of neurons.

In fact, there are different voltage-gated K^+^ currents in neurons, which could be attributable to the expression of several channel types, including the Kv1, Kv2, Kv3 and Kv7 families [22, 42]. It is well known that the action potential is initiated in the axon initial segment (AIS), where the Kv1 channels are particularly dense [43–45]. There are seven members in Kv1 family, which are Kv1.1-Kv1.7 [23, 42]. Some of them have “delayed rectifier” properties (i.e., Kv1.1, Kv1.2, Kv1.5, and Kv1.6), while others exhibit fairly rapid inactivation. Most delayed rectifier Kv1 subunits are sensitive to α-dendrotoxin (α-DTX), such as Kv1.1, Kv1.2, and Kv1.6 [22, 23, 46–48]. These α-DTX-sensitive Kv1 subunits are very active in the subthreshold voltage range, which could powerfully regulate spike threshold and blocking them is able to result in a small but significant negative shift in spike threshold [11, 21–24, 48]. In particular, recent experiments find that blocking the low-threshold Kv1 channel with α-DTX could result in a loss of the inverse relation between spike threshold and d*V*/d*t* in auditory brainstem neurons [15] and layer 2/3 cortex pyramidal neurons [4]. Another α-DTX-sensitive K^+^ current widely present in AIS is the “D-type” K^+^ current, also called D-current [11, 21, 23]. This current also activates at the subthreshold potentials, and has fast activation kinetics as well as very slow inactivation kinetics. Since it is sensitive to α-DTX, the D-current is mediated by channels containing Kv1.1, Kv1.2 and/or Kv1.6. It has been shown that this low-threshold current is also capable of regulating spike threshold, and adjusting it could result in linear depolarization of spike threshold in hippocampal and cortical pyramidal neurons [21, 23, 48, 49]. All these experimental results suggest that the low-threshold K^+^ current in Type II neuron model has a similar functional role in regulating spike threshold dynamics with α-DTX-sensitive Kv1 channels. Unlike Kv1 channels, the medial nucleus of trapezoid body expresses high-threshold K^+^ channels, such as Kv3.1 [50, 51]. These high-threshold K^+^ channels mainly minimize action potential duration and have few modulatory effects on threshold dynamic [46, 50, 51], which may be related to the K^+^ current in our Type I neuron model. Furthermore, Rothman and Manis [52–54] have found that a high density of α-DTX-sensitive current in ventral cochlear nucleus is responsible for phasic firing of type II neurons, while a lower density of α-DTX-sensitive current promotes regular firing of type I cells. This finding further confirms our above inferences.

To generalize our predictions and biophysical explanations, we further examine the effects of altering the voltage-dependency, kinetics or conductance magnitude of Na^+^ and K^+^ currents on threshold dynamic for two cell types. The consequences from each case are all consistent and could be explained by how varying those parameters affect the net current at the threshold potentials. If there is a strong hyperpolarizing net current prior to spike initiation, it will prevent inward Na^+^ from becoming self-sustaining at a fixed voltage and result in a dynamic threshold sensitive to d*V*/d*t*. This suggests that the different spike thresholds in two types of neurons are also the outcomes of the subthreshold competitions between inward and outward currents, which are the same as their spike initiation dynamic [29]. Goldberg et al [24] recently proposed that the dynamic interactions between Kv1 and Na^+^ channels could regulate the spike threshold of fast-spiking cells, which is in agreement with our study. Meanwhile, conceptualizing the spike threshold dependence on d*V*/d*t* as an activation of outward current prior to spike initiation could facilitate the generalization of our results.

Apart from experiments, there are also modeling studies about how outward K^+^ current affects spike threshold. For instance, Shen et al [48] adopt a Hodgkin-Huxley-like model based on biophysical data to show that the blockade of Kv1.2 channels would reduce the threshold potential of striatal medium spiny cells; Wester and Contreras [20] recently use a three-compartment model to show that hyperpolarizing K^+^ activation to make it activate prior to spike initiation is sufficient to produce an inverse relation between d*V*/d*t* and spike threshold. All these modelling predictions are in agreement with our results. Besides, Prescott et al [29] have provided a generalizable explanation of biophysical basis for two types of excitability based on the two-dimensional model. However, the specific spike threshold for Type I and Type II neurons is not explored in that study. By analyzing the interactions of membrane currents at the perithresholds, this work further associates the biophysical properties of two cell types to their spike threshold. As a consequence, our biophysical explanation of spike threshold is not specific to one category of neuron, but should be generalized to all neurons.

Finally, the low-threshold K^+^ current would also influence how neuron encodes synaptic input into spiking output, such as, enhancing coincidence detection [55] or improving the temporal precision of weak signals [56]. The dynamic spike threshold has been shown to produce the similar functional roles on neural coding [3, 4, 6, 7, 20, 27, 28]. Then, characterizing how outward K^+^ current modulates the spike threshold dynamics of two cell types could provide a greater insight into how this kind of current participates in neural coding.

### Threshold dynamics of Type III neurons

**Fig 11:**
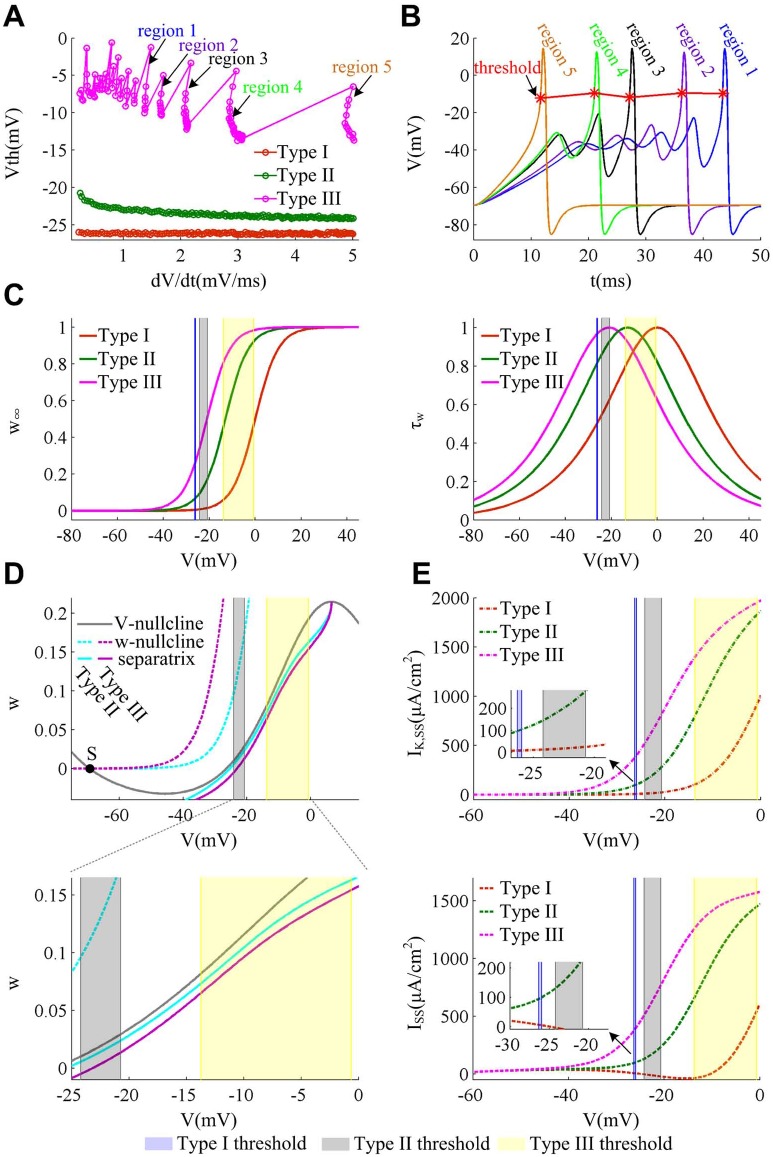
Dynamical and biophysical basis of spike threshold for Type III excitability. (A) Spike threshold for three types of excitability within a range of d*V*/d*t* (from 0.2mV/ms to 5.0mV/ms) produced by the minimal model. (B) Sample spikes of Type III neuron in the five regions in (A). Since d*V*/d*t* is successively increased from region 1 to region 5, the corresponding ramp slope *K* used to evoke it should also increase, which is 2.9μA/(cm^2^·ms), 3.2μA/(cm^2^·ms), 4.1μA/(cm^2^·ms), 4.3μA/(cm^2^·ms) and 5.8μA/(cm^2^·ms), in that order. For slower ramps, there are small oscillations in membrane potential (i.e., the delayed loss of stability) prior to spike initiation. As slope *K* increase, this delayed phenomenon gets shorter, which even disappears for some higher slope *K*, such as region 5. (C) Activation curves and time constant of K^+^ current for three cell types. From Type I to Type III, the half-activation potential of *w*_∞_ is 0mV, -13mV and -21mV, and simultaneously the time constant *τ*_*w*_ also shifts to the hyperpolarized potential. (D) Phase portraits and corresponding separatrix for Type II and Type III neurons. In the absence of current ramp, there is only one stable focus (black dot) between *V*- and *w*-nullclines for both cell types. However, Type III separatrix is at the right side of Type II, which corresponds to a depolarized spike threshold. “S” stands the intersection is stable. (E) *I*_K,SS_ and *I*_SS_ at the subthresholds. The blue region is the spike threshold of Type I, the gray is Type II and the yellow is Type III.

Except for Type I and Type II excitability, there is another neural excitability, i.e., Type III excitability, defined by Hodgkin [2, 29, 41]. This excitability typically generates single spike in response to a pulse of current, which is very common in sensory pathway [2, 29, 41]. Prescott et al [29] have shown that the outward K^+^ current in Type III neuron is the most easily to activate in Hodgkin’s three types of excitability. According to our prediction, one can infer that Type III excitability should have a more depolarized spike threshold as well as a more pronounced relationship between threshold and d*V*/d*t* compared to the other two types. To test this inference, we use the minimal model to further investigate the Type III spike threshold within a range of d*V*/d*t*, which is summarized in Fig 11.

In Type III neuron, the half-activation voltage of *w*_∞_ is hyperpolarized by -21mV than Type I (left panel, Fig 11C), which leads the outward K^+^ current to activate at an even more hyperpolarized potential than Type II neuron (top panel, Fig 11E). Then, the Type III net current is much larger than Type II at its threshold potentials (bottom panel, Fig 11E). From Fig 11A, we can observe that the Type III neuron indeed has a more depolarized threshold than Type II, which is in accordance with our prediction and biophysical explanation. However, its spike threshold shows large fluctuations as d*V*/d*t* increases. This arises from the small oscillations before spike initiation (Fig 11B), which leads to big errors when we compute d*V*/d*t*. This phenomenon is called the delayed loss of stability, which is first discovered by Shishkova [2, 57]. It is ubiquitous in the stimulations of smooth dynamical system near Hopf bifurcation. In the case of Type III excitability (i.e., β_*w*_ = -21mV), the minimal model generates Hopf bifurcation in a very strong current (87.25μA/cm^2^), which conforms with the above-mentioned conditions.

The mechanism for this delayed phenomenon is quite simple. Before spike initiation, the state of the system is attracted by the stable focus. When current ramp slowly drives *V* across threshold, the state of the system is infinitesimally close to the equilibrium. Then, its trajectory takes a long time to diverge from it, which results in the small oscillations. The longer the trajectory takes to converge to the equilibrium, the longer it takes to diverge from it [2, 57]. So the delayed phenomenon is more noticeable in the case of low ramp slope. In fact, the Type II excitability could also generate the delay when ramp slope is very low, that is why the d*V*/d*t* range in Figs 8–10 is narrower than that in Fig 1. But the delayed phenomenon in Type II excitability is shorten and less obvious than Type III. This is because the Type III separatrix lies on the right side of Type II (Fig 11D), and meanwhile its *V* trajectories varies in a larger scale along the *w*-axis (since *w*_∞_ is much larger in Type III neuron than Type II at its corresponding threshold potentials). Then, with the same slow current ramp, Type II *V* trajectory could quickly pass through the near separatrix without delay, whereas Type III needs a long time to get across the remote separatrix with delay. To eliminate the large fluctuations and get a smooth spike threshold curve in Type III excitability, we could use curve fitting to deal with the obtain spike thresholds or use filtering method to smooth the *V* trajectory. Whatever the approach, we are supposed to obtain a pronounced inverse relation between spike threshold and d*V*/d*t* (data is not shown). Thus, our predictions and biophysical explanations are also practicable in the case of Type III excitability, which in turn facilitates the generalization of our conclusion.

### Limitations of the biophysical model and technical considerations

Our stimulation, which is highly simplified, only contains two ionic currents in a single-compartment model. In central neurons, the spike is initiated at the AIS while the spike threshold is measured at the soma *in vivo*, which could be an artificial cause of threshold variability [3, 18, 58, 59]. Then, there is a debate about the validity of using single-compartment model to explore the spike threshold for cortical neurons. But whether distal spike initiation accounts for the inverse relation between threshold and d*V*/d*t* is still unclear. More important, recent modelling studies [3, 20] find that although the inverse relation is more pronounced at the AIS than at the soma, the distal initiation cannot be the dominant cause of threshold variability, which is also unable to account for the threshold dynamics.

The spike threshold of the selected model is in the -35mV to -15mV range, which is far more depolarized than that observed in complex or realistic neurons. This is due to the simplified form of our model as well as the selected values of model parameters. However, these differences of the threshold value induced by model parameters cannot essentially alter the dependence of spike threshold on d*V*/d*t* preceding the spike. So we could use simple model to qualitatively characterize the spike threshold in Type I and Type II neurons within a range of d*V*/d*t*. The reason that we use such a simple model is because it not only involves the essential ionic mechanisms to generate a spike, but also excludes the extraneous details. Then, we could use phase plane analysis to explore how the different spike threshold dynamics arise in two cell types, and we could also determine what intrinsic properties of membrane currents are crucial to these dynamics and why. Meanwhile, Platkiewicz and Brette [3] have shown that it is feasible to using a single-compartment model to investigate the threshold variability and its biophysical basis, which is also confirmed by Wester and Contreras [20] in their stimulations. Moreover, unlike Hodgkin-Huxley model, the two-dimensional neuron in this study does not include Na^+^ inactivation. Since the upstroke of the spike is mainly generated by Na^+^ channel, its inactivation is generally regarded as an important factor regulating spike threshold [3, 6–8, 17–20]. But more and more studies find that the low-threshold outward K^+^ current at the AIS could also powerfully modulate spike threshold. In particular, Wester and Contreras [20] recently report that hyperpolarizing K^+^ current activation voltage, even in the absence of Na^+^ inactivation, is also sufficient to produce the inverse relation between threshold and d*V*/d*t*. Thus, using the simple model that does not involve Na^+^ inactivation to explore threshold dynamic in Type I and Type II neurons is feasible. Even so, one of our further works is to test our predictions and biophysical explanations in a more biophysically realistic model as well as in neurophysiological experiments.

There are also some limitations of the threshold measuring method in present study. First, although this method is directly related to neurophysiological experiments, the threshold potential obtained by it is very specific to current ramp, which would not generalize to other stimulus since the threshold has been shown to depend on neuronal spiking history [3–7, 16, 20]. Second, its predictive power is less than the threshold equation proposed by Platkiewicz and Brette [3, 19]. Further, the experiments do not commonly use this method to determine threshold voltage due to its complex operations. *In vivo*, the spike threshold is usually defined as the voltage at which d*V*/d*t* exceeds an empirical value [6, 7, 16, 18, 58], or as the first peak in the third derivative of the recorded action potential [9]. But both of them are purely empirical, as their criterions for threshold detection are just above the maximal level of d*V*/d*t* seen during the spontaneous subthreshold activity. Unlike them, the approach used in this study allows one to describe the quantitative relation between spike threshold and d*V*/d*t* in a precise manner, especially for the complex neurons modeled by NEURON. Its utility has been attested by Wester and Contreras [20], and we hope our study could lead to increased applications of this method in the future theoretical research of spike threshold.

In summary, the different spike threshold in Type I and Type II excitability results from their distinct subthreshold properties of inward and outward currents. The activation of outward K^+^ current prior to spike initiation is a major factor that contributes to the dynamic threshold in Type II neuron. Our study provides a fundamental connection that links biophysical properties, spike threshold and spike initiation dynamics. Identifying the functional significance of biophysical properties in spike threshold could contribute to uncover how neuron encodes synaptic inputs as a train of action potentials, which is also conducive to reveal the relevant mechanism of spike adaptation, feature selectivity, temporal sensitivity, reliability and precision, or even the modulatory effects of spike time by subthreshold electric fields [60–62].

## Materials and Methods

### Two-dimensional neuron model

We use a modified Morris-Lecar model proposed by Prescott et al [29] to stimulate Type I and Type II excitability. This is a two-dimensional neuron model, which incorporates two variables, i.e., membrane potential *V* and activation gate *w* for K^+^ channel. The change of membrane potential *V* obeys the following current balance equation

$$\text{C}\frac{\text{d}V}{\text{d}t}=I_{\text{S}}-I_{\text{Na}}-I_{\text{K}}-I_{\text{L}}$$ (1)

where *I*_S_ is the injected current and C = 2.0μF/cm^2^ is the membrane capacitance. The membrane currents are comprised of a spike-generating Na^+^ current *I*_Na_, a delayed rectifier K^+^ current *I*_K_ and a leak current *I*_L_, which obey the following equations

$$I_{\text{Na}}={\bar{\text{g}}}_{\text{Na}}m_{\infty }(V)(V-{\text{E}}_{\text{Na}})$$ (2)

$$I_{\text{K}}={\bar{\text{g}}}_{\text{K}}w(V-{\text{E}}_{\text{K}})$$ (3)

$$I_{\text{L}}={\text{g}}_{\text{L}}(V-{\text{E}}_{\text{L}})$$ (4)

In eqs (2)–(4), ${\bar{\text{g}}}_{\text{Na}}\text{=20}\text{mS}/{\text{cm}}^{\text{2}}$, ${\bar{\text{g}}}_{\text{K}}\text{=20}\text{mS}/{\text{cm}}^{\text{2}}$ and g_L_ = 2mS/cm^2^ are the maximum conductance values for Na^+^, K^+^ and leak currents, respectively. Their corresponding reversal potential values are E_Na_ = 50mV, E_K_ = -100mV and E_L_ = -70mV. The variable *w* denotes the activation gate for K^+^ channel, which evolves according to

$$\frac{\text{d}w}{\text{d}t}=\varphi_{w}\frac{w_{\infty }(V)-w}{\tau_{w}(V)}$$ (5)

where φ_*w*_ = 0.15 is the scaling factor for variable *w*. *w*_∞_(*V*) and *τ*_*w*_(*V*) are the steady-state activation and time constant of K^+^ current. Further, *m*_∞_(*V*) in eq (2) represents the steady-state activation of Na^+^ current. These three terms are all functions of membrane potential *V*, which are described by

$$\begin{array}{l} m_{\infty }(V)=0.5\left[1+\text{tanh}\left(\frac{V-\beta_{m}}{\gamma_{m}}\right)\right]\\ w_{\infty }(V)=0.5\left[1+\text{tanh}\left(\frac{V-\beta_{w}}{\gamma_{w}}\right)\right]\\ \tau_{w}(V)=1/\text{cosh}\left(\frac{V-\beta_{w}}{2\gamma_{w}}\right) \end{array}$$ (6)

where β*m* = -1.2mV, γ_*m*_ = 18mV and γ_*m*_ = 10mV.

Prescott et al [29] showed that varying parameter β_*w*_ is capable of converting the neuron between Type I and Type II excitability. For β_*w*_ = 0mV, the neuron exhibits Type I excitability with a continuous *f*-*I* curve. For β_*w*_ = -13mV, it exhibits Type II excitability with a discontinuous *f*-*I* curve. If further decrease this parameter, such as β_*w*_ = -21mV, the model will exhibit Type III excitability, which can only generate an action potential in the biophysically relevant stimulus range. Moreover, varying other parameters, such as, β_*m*_, γ_*m*_, ${\bar{\text{g}}}_{\text{Na}}$ or even ${\bar{\text{g}}}_{\text{K}}$ could also switch neuron from Type I to Type II excitability.

### Three-dimensional neuron model

By dividing *I*_K_ into two different component parts, i.e., *I*_K,dr_ and *I*_Sub_, Prescott et al [29] successfully converted the model from two-dimensional to three-dimensional and make a more biophysically realistic neuron. The current balance equation for this model is

$$\text{C}\frac{\text{d}V}{\text{d}t}=I_{\text{S}}-I_{\text{Na}}-I_{\text{K,dr}}-I_{\text{sub}}-I_{\text{L}}$$ (7)

where $I_{\text{K,dr}}={\bar{\text{g}}}_{\text{K,dr}}y(V-{\text{E}}_{\text{K}})$ is a delayed rectifier K^+^ current and $I_{\text{sub}}={\bar{\text{g}}}_{\text{sub}}z(V-{\text{E}}_{\text{sub}})$ stands a subthreshold current. The gating variable *y* and *z* are governed by

$$\begin{array}{l} \frac{\text{d}y}{\text{d}t}=\varphi_{y}\frac{y_{\infty }(V)-y}{\tau_{y}(V)}\\ \frac{\text{d}z}{\text{d}t}=\varphi_{z}\frac{z_{\infty }(V)-z}{\tau_{z}(V)} \end{array}$$ (8)

The voltage-dependent steady-state function and time constant for variable *y* and *z* are

$$\begin{array}{l} y_{\infty }(V)=0.5\left[1+\text{tanh}\left(\frac{V-\beta_{y}}{\gamma_{y}}\right)\right],\;\tau_{y}(V)=1/\text{cosh}\left(\frac{V-\beta_{y}}{2\gamma_{y}}\right)\\ z_{\infty }(V)=0.5\left[1+\text{tanh}\left(\frac{V-\beta_{z}}{\gamma_{z}}\right)\right],\;\tau_{z}(V)=1/\text{cosh}\left(\frac{V-\beta_{z}}{2\gamma_{z}}\right) \end{array}$$ (9)

*I*_Sub_ is either inward or outward, which is dependent on its reversal potential E_sub_. For E_sub_ = E_Na_ = 50mV, ${\bar{\text{g}}}_{\text{sub}}=3\text{mS}/{\text{cm}}^{\text{2}}$ and φ_z_ = 0.5, *I*_sub_ is inward and the three-dimensional neuron exhibits Type I excitability, which could generate arbitrarily low frequency spiking. For E_sub_ = E_K_ = -100mV, ${\bar{\text{g}}}_{\text{sub}}=2\text{mS}/{\text{cm}}^{\text{2}}$ and φ_z_ = 0.15, *I*_sub_ is outward and the neuron exhibits Type II excitability, which fails to maintain repetitive spiking below a critical frequency. Further, β_z_ = -21mV, γ_z_ = 15mV, β_y_ = -10mV, γ_y_ = 10mV, φ_y_ = 0.15, ${\bar{\text{g}}}_{\text{K,dr}}=\text{20}\text{mS}/{\text{cm}}^{\text{2}}$, and the other parameters are all identical with those in the two-dimensional model.

### Stimulation method to determine spike threshold

**Fig 12:**
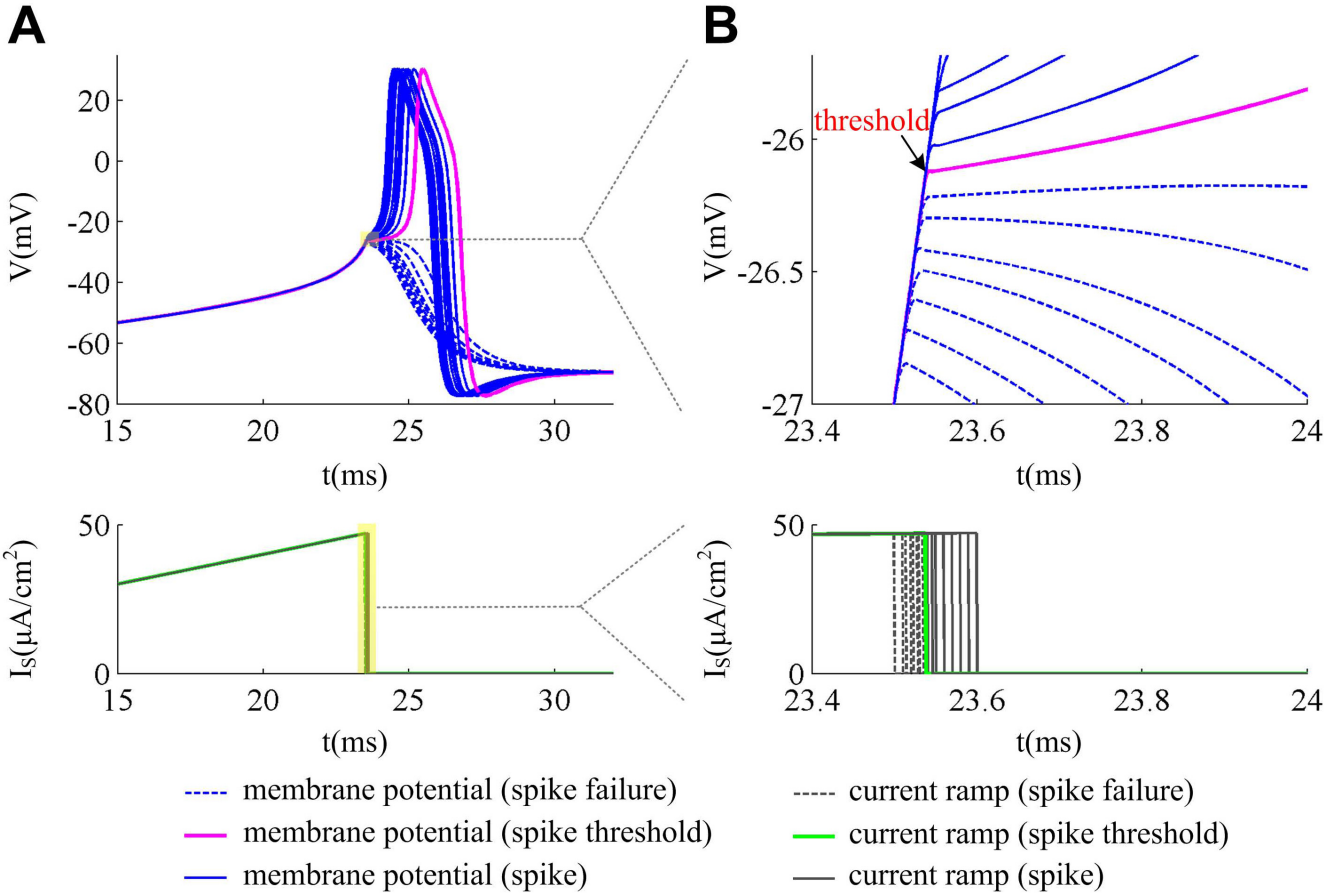
Stimulation method for determining spike threshold. The neuron is stimulated by current ramp *I*_S_, and the ramp slope *K* is used to control d*V*/d*t*. The ramp duration is empirically stepwise increased, such that each step could result in a 0.1mV depolarization in membrane potential. (A) gives neuronal responses in the vicinity of spike threshold, and (B) is the enlarge views of the yellow regions in (A). We define spike threshold as the membrane potential at the time of *I*_S_ offset. Then, 0.1mV hyperpolarized to this potential is suprathreshold which results in a spike, while 0.1mV depolarized to this potential is subthreshold which leads to spike failure.

Our study adopts a novel method proposed by Wester and Contreras [20] to precisely determine the spike threshold of neuron model. We inject ramps of current *I*_S_ with different slopes *K* into neuron to control the rate of membrane potential depolarization, i.e., d*V*/d*t*. For a given slope *K*, the current ramp with less stimulus duration could not evoke action potential, only if that with sufficient duration could trigger spikes in the neuron (Fig 12). Thus, we stepwise increase the ramp duration empirically, such that each step could result in an additional 0.1mV depolarization in membrane potential until an action potential is evoked (Fig 12B). The spike threshold is defined as the *V* at the time of current ramp offset (Fig 12B), by which the spike initiation is due to the activation of Na^+^ current purely and has nothing to do with the current ramp. Then, 0.1mV depolarized to this *V* is suprathreshold and could lead to a spike, while 0.1mV hyperpolarized to this *V* is subthreshold for spike initiation. Through this method, we could not only precisely measure spike threshold (with a precision less than 0.1mV) and stimulus duration, but also investigate the accurate properties of the ionic currents and gating variables at the spike threshold.

### Numerical stimulation

The numerical simulations of the whole system are run with MATLAB (http://www.mathworks.com), and the numerical phase plane analysis is performed by using XPPAUT [63] (http://www.math.pitt.edu/~bard/xpp/xpp.html).

## References

[1] Dayan P, Abbott LF (2005) Theoretical neuroscience: computational and mathematical modeling of neural systems. London: The MIT Press.

[2] Izhikevich EM (2005) Dynamical systems in neuroscience: The geometry of excitability and bursting. London: The MIT Press.

[3] Platkiewicz J, Brette R (2011) Impact of fast sodium channel inactivation on spike threshold dynamics and synaptic integration. PLoS Comput Biol 7: e1001129 10.1371/journal.pcbi.1001129 21573200 PMC3088652

[4] Higgs MH, Spain WJ (2011) Kv1 channels control spike threshold dynamics and spike timing in cortical pyramidal neurones. Journal of Physiology 589: 5125–5142. 10.1113/jphysiol.2011.216721 21911608 PMC3225669

[5] Fontaine B, Peña JL, Brette R (2014) Spike-threshold adaptation predicted by membrane potential dynamics *in vivo* . PLoS Comput Biol 10: e1003560 10.1371/journal.pcbi.1003560 24722397 PMC3983065

[6] Azouz R, Gray CM (2000) Dynamic spike threshold reveals a mechanism for synaptic coincidence detection in cortical neurons *in vivo* . Proc Natl Acad Sci U S A 97: 8110–8115. 10859358 10.1073/pnas.130200797 PMC16678

[7] Azouz R, Gray CM (2003) Adaptive coincidence detection and dynamic gain control in visual cortical neurons *in vivo* . Neuron 37: 513–523. 12575957 10.1016/s0896-6273(02)01186-8

[8] Wilent WB, Contreras D (2005) Stimulus-dependent changes in spike threshold enhance feature selectivity in rat barrel cortex neurons. J Neurosci 25: 2983–2991. 15772358 10.1523/JNEUROSCI.4906-04.2005 PMC6725135

[9] Henze DA, Buzsaki G (2001) Action potential threshold of hippocampal pyramidal cells *in vivo* is increased by recent spiking activity. Neuroscience 105: 121–130. 11483306 10.1016/s0306-4522(01)00167-1

[10] Hodgkin AL, Huxley AF (1952) A quantitative description of membrane current and its application to conduction and excitation in nerve. J Physiol 117: 500–544. 12991237 10.1113/jphysiol.1952.sp004764 PMC1392413

[11] Storm JF (1988) Temporal integration by a slowly inactivating K+ current in hippocampal neurons. Nature 336: 379–381. 3194020 10.1038/336379a0

[12] Cardin JA, Kumbhani RD, Contreras D, Palmer LA (2010) Cellular mechanisms of temporal sensitivity in visual cortex neurons. J Neurosci 30: 3652–3662. 10.1523/JNEUROSCI.5279-09.2010 20219999 PMC2880457

[13] Schlue WR, Richter DW, Mauritz KH, Nacimiento AC (1974) Responses of cat spinal motoneuron somata and axons to linearly rising currents. J Neurophysiol 37: 303–309. 4815207 10.1152/jn.1974.37.2.303

[14] Escabí MA, Nassiri R, Miller LM, Schreiner CE, Read HL (2005) The contribution of spike threshold to acoustic feature selectivity, spike information content, and information throughput. J Neurosci 25: 9524–9534. 16221863 10.1523/JNEUROSCI.1804-05.2005 PMC6725702

[15] Ferragamo MJ, Oertel D (2002) Octopus cells of the mammalian ventral cochlear nucleus sense the rate of depolarization. J Neurophysiol 87: 2262–2270. 11976365 10.1152/jn.00587.2001

[16] Muñoz F., Fuentealba P. (2012) Dynamics of action potential initiation in the GABAergic thalamic reticular nucleus in vivo. PLoS ONE 7: e30154 10.1371/journal.pone.0030154 22279567 PMC3261188

[17] Hu W, Tian C, Li T, Yang M, Hou H, Shu Y (2009) Distinct contributions of Na(v)1.6 and Na(v)1.2 in action potential initiation and backpropagation. Nature Neurosci 12: 996–1002. 10.1038/nn.2359 19633666

[18] Naundorf B, Wolf F, Volgushev M (2006) Unique features of action potential initiation in cortical neurons. Nature 440: 1060–1063. 16625198 10.1038/nature04610

[19] Platkiewicz J, Brette R (2010) A threshold equation for action potential initiation. PLoS Comput Biol 6: e1000850 10.1371/journal.pcbi.1000850 20628619 PMC2900290

[20] Wester JC, Contreras D (2013) Biophysical mechanism of spike threshold dependence on the rate of rise of the membrane potential by sodium channel inactivation or subthreshold axonal potassium current. J Comput Neurosci 35(1): 1–17. 10.1007/s10827-012-0436-2 23344915 PMC3683126

[21] Bekkers JM, Delaney AJ (2001) Modulation of excitability by alpha-dendrotoxin-sensitive potassium channels in neocortical pyramidal neurons. J Neurosci 21: 6553–6560. 11517244 10.1523/JNEUROSCI.21-17-06553.2001 PMC6763106

[22] Guan D, Lee JC, Higgs MH, Spain WJ, Foehring RC (2007) Functional roles of Kv1 channels in neocortical pyramidal neurons. J Neurophysiol 97: 1931–40. 17215507 10.1152/jn.00933.2006

[23] Dodson PD, Barker MC, Forsythe ID (2002) Two heteromeric Kv1 potassium channels differentially regulate action potential firing. J Neurosci 22: 6953–61. 12177193 10.1523/JNEUROSCI.22-16-06953.2002 PMC6757903

[24] Goldberg EM, Clark BD, Zagha E, Nahmani M, Erisir A, Rudy B (2008) K+ channels at the axon initial segment dampen near-threshold excitability of neocortical fast-spiking GABAergic interneurons. Neuron 58: 387–400. 10.1016/j.neuron.2008.03.003 18466749 PMC2730466

[25] Priebe NJ, Ferster D (2008) Inhibition, spike threshold, and stimulus selectivity in primary visual cortex. Neuron 57: 482–497. 10.1016/j.neuron.2008.02.005 18304479

[26] Fontaine B, MacLeod KM, Lubejko ST, Steinberg LJ, Köppl C, Peña JL (2014) Emergence of band-pass filtering through adaptive spiking in the owl’s cochlear nucleus. Journal of Neurophysiology 112: 430–445. 10.1152/jn.00132.2014 24790170 PMC4064407

[27] Kuba H, Ishii TM, Ohmori H (2006) Axonal site of spike initiation enhances auditory coincidence detection. Nature 444: 1069–1072. 17136099 10.1038/nature05347

[28] Fontaine B, Benichoux V, Joris PX, Brette R (2013) Predicting spike timing in highly synchronous auditory neurons at different sound levels. Journal of Neurophysiology 110: 1672–1688. 10.1152/jn.00051.2013 23864375 PMC4042421

[29] Prescott SA, De Koninck Y, Sejnowski TJ (2008) Biophysical basis for three distinct dynamical mechanisms of action potential initiation. PLoS Comput Biol 4(10): e1000198 10.1371/journal.pcbi.1000198 18846205 PMC2551735

[30] St-Hilaire M, Longtin A (2004) Comparison of coding capabilities of Type I and Type II neurons. J Comput Neurosci 16(3): 299–313. 15114051 10.1023/B:JCNS.0000025690.02886.93

[31] Mato G, Samengo I (2008) Type I and type II neuron models are selectively driven by differential stimulus features. Neural Comput 20(10): 2418–40. 10.1162/neco.2008.10-07-632 18439139

[32] Izhikevich EM (2000) Neural excitability, spiking and bursting. Int. J. Bifurcation Chaos 10: 1171–1266.

[33] Drion G, Franci A, Seutin V, Sepulchre R (2012) A novel phase portrait for neuronal excitability. PLoS One 7: e41806 10.1371/journal.pone.0041806 22905107 PMC3414513

[34] FitzHugh R (1960) Threshold and plateaus in the Hodgkin-Huxley nerve equations. The Journal of General Physiology 43: 867–896. 13823315 10.1085/jgp.43.5.867 PMC2195039

[35] FitzHugh R (1961) Impulses and physiological states in theoretical models of nerve membrane. Biophys J 1: 445–466. 19431309 10.1016/s0006-3495(61)86902-6 PMC1366333

[36] Llina´s RR (1988) The intrinsic electrophysiological properties of mammalian neurons: insights into central nervous system function. Science 242: 1654–1664. 3059497 10.1126/science.3059497

[37] Connors BW, Gutnick MJ (1990) Intrinsic firing patterns of diverse neocortical neurons. Trends Neurosci 13: 99–104. 1691879 10.1016/0166-2236(90)90185-d

[38] Yi GS, Jiang W, Wei XL, Tsang KM, Chan WL, Deng B (2014) Neuronal spike initiation modulated by extracellular electric fields. PLoS One 9: e97481 10.1371/journal.pone.0097481 24873827 PMC4038635

[39] Yi GS, Wang J, Tsang KM, Wei XL, Deng B, Han CX (2015) Spike-frequency adaptation of a two-compartment neuron modulated by extracellular electric fields. Biological Cybernetics. 10.1007/s00422-014-0642-2 25652337

[40] Prescott SA, Ratté S, De Koninck Y, Sejnowski TJ (2008) Pyramidal neurons switch from integrators in vitro to resonators under *in vivo*-like conditions. J Neurophysiol 100: 3030–3042. 10.1152/jn.90634.2008 18829848 PMC2604842

[41] Meng X, Huguet G, Rinzel J (2012) Type III excitability, slope sensitivity and coincidence detection. Discrete Contin Dyn Syst Ser A 32(8): 2729–2757. 23667306 10.3934/dcds.2012.32.2729 PMC3647375

[42] Hille B (2001) Ion channels of excitable membranes. Sunderland: Sinauer.

[43] Debanne D, Campanac E, Bialowas A, Carlier E, Alcaraz G (2011) Axon physiology. Physiological Reviews 91: 555–602. 10.1152/physrev.00048.2009 21527732

[44] Bender KJ, Trussell LO (2012) The physiology of the axon initial segment. Annual Review of Neuroscience 35: 249–265. 10.1146/annurev-neuro-062111-150339 22443507

[45] Kole MH, Stuart GJ (2012) Signal processing in the axon initial segment. Neuron 73: 235–247. 10.1016/j.neuron.2012.01.007 22284179

[46] Brew HM, Forsythe ID (1995) Two voltage-dependent K+ conductances with complementary functions in postsynaptic integration at a central auditory synapse. J Neurosci 15: 8011–8022. 8613738 10.1523/JNEUROSCI.15-12-08011.1995 PMC6577951

[47] Harvey AL, Robertson B (2004) Dendrotoxins: structureactivity relationships and effects on potassium ion channels. Current Medicinal Chemistry 11: 3065–3072. 15579000 10.2174/0929867043363820

[48] Shen W, Hernandez-Lopez S, Tkatch T, Held JE, Surmeier DJ (2004) Kv1.2-containing K+ channels regulate subthreshold excitability of striatal medium spiny neurons. Journal of Neurophysiology 91: 1337–1349. 13679409 10.1152/jn.00414.2003

[49] Shu Y, Yu Y, Yang J, McCormick DA (2007) Selective control of cortical axonal spikes by a slowly inactivating K+ current. Proceedings of the National Academy of Sciences of the United States of America 104: 11453–11458. 17581873 10.1073/pnas.0702041104 PMC2040919

[50] Wang LY, Gan L, Forsythe ID, Kaczmarek LK (1998) Contribution of the Kv3.1 potassium channel to high-frequency firing in mouse auditory neurones. J Physiol 509: 183–194. 9547392 10.1111/j.1469-7793.1998.183bo.x PMC2230948

[51] Rudy B, McBain CJ (2001) Kv3 channels: voltage-gated K+ channels designed for high-frequency repetitive firing. Trends Neurosci 24: 517–526. 11506885 10.1016/s0166-2236(00)01892-0

[52] Rothman JS, Manis PB (2003) Differential expression of three distinct potassium currents in the ventral cochlear nucleus. J Neurophysiol 89: 3070–3082. 12783951 10.1152/jn.00125.2002

[53] Rothman JS, Manis PB (2003) Kinetic analyses of three distinct potassium currents in the ventral cochlear nucleus. J Neurophysiol 89: 3083–3096. 12783952 10.1152/jn.00126.2002

[54] Rothman JS, Manis PB (2003) The roles potassium currents play in regulating the electrical activity of ventral cochlear nucleus neurons. J Neurophysiol 89: 3097–3113. 12783953 10.1152/jn.00127.2002

[55] Svirskis G, Kotak V, Sanes DH, Rinzel J (2004) Sodium along with low-threshold potassium currents enhance coincidence detection of subthreshold noisy signals in MSO neurons. J Neurophysiol 91(6): 2465–73. 14749317 10.1152/jn.00717.2003 PMC3683536

[56] Svirskis G, Kotak V, Sanes DH, Rinzel J (2002) Enhancement of signal-to-noise ratio and phase locking for small inputs by a low-threshold outward current in auditory neurons. J Neurosci 22(24): 11019–25. 12486197 10.1523/JNEUROSCI.22-24-11019.2002 PMC3677217

[57] Shishkova MA (1973) Investigation of a system of differential equations with a small parameter in the highest derivatives. Dokl. Akad. Nauk SSSR 209: 576–579.

[58] Yu Y, Shu Y, McCormick DA (2008) Cortical action potential backpropagation explains spike threshold variability and rapid-onset kinetics. J Neurosci 28: 7260–7272. 10.1523/JNEUROSCI.1613-08.2008 18632930 PMC2664555

[59] Brette R (2013) Sharpness of spike initiation in neurons explained by compartmentalization. PLoS Comput Biol 9: e1003338 10.1371/journal.pcbi.1003338 24339755 PMC3854010

[60] Yi GS, Wang J, Wei XL, Tsang KM, Chan WL, Deng B, et al (2014) Exploring how extracellular electric field modulates neuron activity through dynamical analysis of a two-compartment neuron model. Journal of Computational Neuroscience 36: 383–399. 10.1007/s10827-013-0479-z 24057225

[61] Reato D, Rahman A, Bikson M, Parra LC (2010) Low-intensity electrical stimulation affects network dynamics by modulating population rate and spike timing. J Neurosci 30: 15067–15079. 10.1523/JNEUROSCI.2059-10.2010 21068312 PMC3500391

[62] Yi GS, Wang J, Wei XL, Bin Deng, Tsang KM, Chan WL, et al (2014) Effects of extremely low-frequency magnetic fields on the response of a conductance-based neuron model. International Journal of Neural Systems 24: 1450007 10.1142/S0129065714500075 24344694

[63] Ermentrout B (2002) Simulating, Analyzing, and Animating Dynamical Systems: A Guide to Xppaut for Researchers and Students. Philadelphia: SIAM.

